# Microwave-Mediated, Catalyst-Free Synthesis of 1,2,4-Triazolo[1,5-*a*]pyridines from Enaminonitriles

**DOI:** 10.3390/molecules29040894

**Published:** 2024-02-18

**Authors:** Kwanghee Lee, Young-Ah Kim, Chanhyun Jung, Jaeuk Sim, Shanmugam Rajasekar, Jae-Hwan Kwak, Mayavan Viji, Jae-Kyung Jung

**Affiliations:** College of Pharmacy and Medicinal Research Center (MRC), Chungbuk National University, Cheongju 28160, Republic of Korea; kwanghee93@naver.com (K.L.); rladuddk97@naver.com (Y.-A.K.); cksgus9823@naver.com (C.J.); simprog@naver.com (J.S.); rajasekarsrkv@gmail.com (S.R.); jhkwak@chungbuk.ac.kr (J.-H.K.)

**Keywords:** enaminonitrile, 1,2,4-triazolo[1,5-*a*]pyridine, microwave irradiation, tandem reaction, acyl hydrazide

## Abstract

A catalyst-free, additive-free, and eco-friendly method for synthesizing 1,2,4-triazolo[1,5-*a*]pyridines under microwave conditions has been established. This tandem reaction involves the use of enaminonitriles and benzohydrazides, a transamidation mechanism followed by nucleophilic addition with nitrile, and subsequent condensation to yield the target compound in a short reaction time. The methodology demonstrates a broad substrate scope and good functional group tolerance, resulting in the formation of products in good-to-excellent yields. Furthermore, the scale-up reaction and late-stage functionalization of triazolo pyridine further demonstrate its synthetic utility. A plausible reaction pathway, based on our findings, has been proposed.

## 1. Introduction

**Figure 1:**
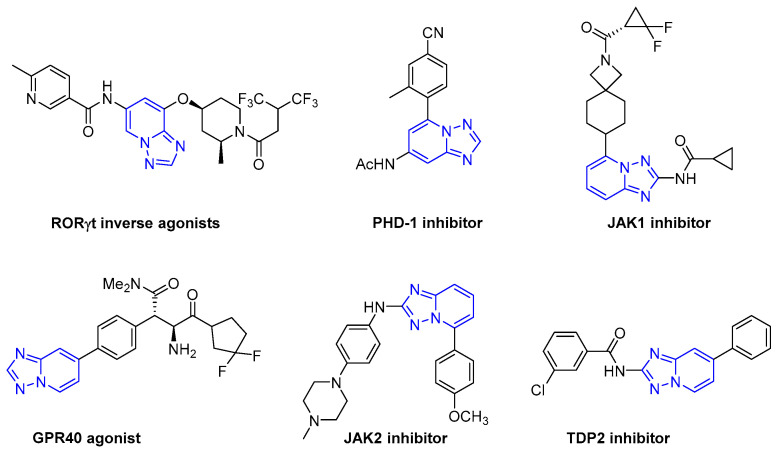
Representative examples of bioactive 1,2,4-triazolo[1,5-*a*]pyridines.

The synthesis of heterocyclic compounds holds enormous applications in medicinal and pharmaceutical chemistry [1]. Nitrogen-containing heterocyclic compounds are found in numerous natural products exhibiting immense biological activities [2]. Developing sustainable methodologies for the synthesis of fused heterocyclic compounds is an actively pursued area of research. 1,2,4-triazolo[1,5-*a*]pyridine, with a bridge-headed nitrogen atom, is usually found in medicinal and biologically active compounds [3,4,5,6]. It exhibits numerous activities, including acting as RORγt inverse agonists [7], PHD-1 [8], JAK1 [9], and JAK2 inhibitors [10] (Figure 1). Also, these compounds are utilized in the treatment of cardiovascular disorders [11], type 2 diabetes [12], and hyperproliferative disorders [13]. Additionally, these types of compounds have various applications in the material sciences fields as well [14].

**Scheme 1:**
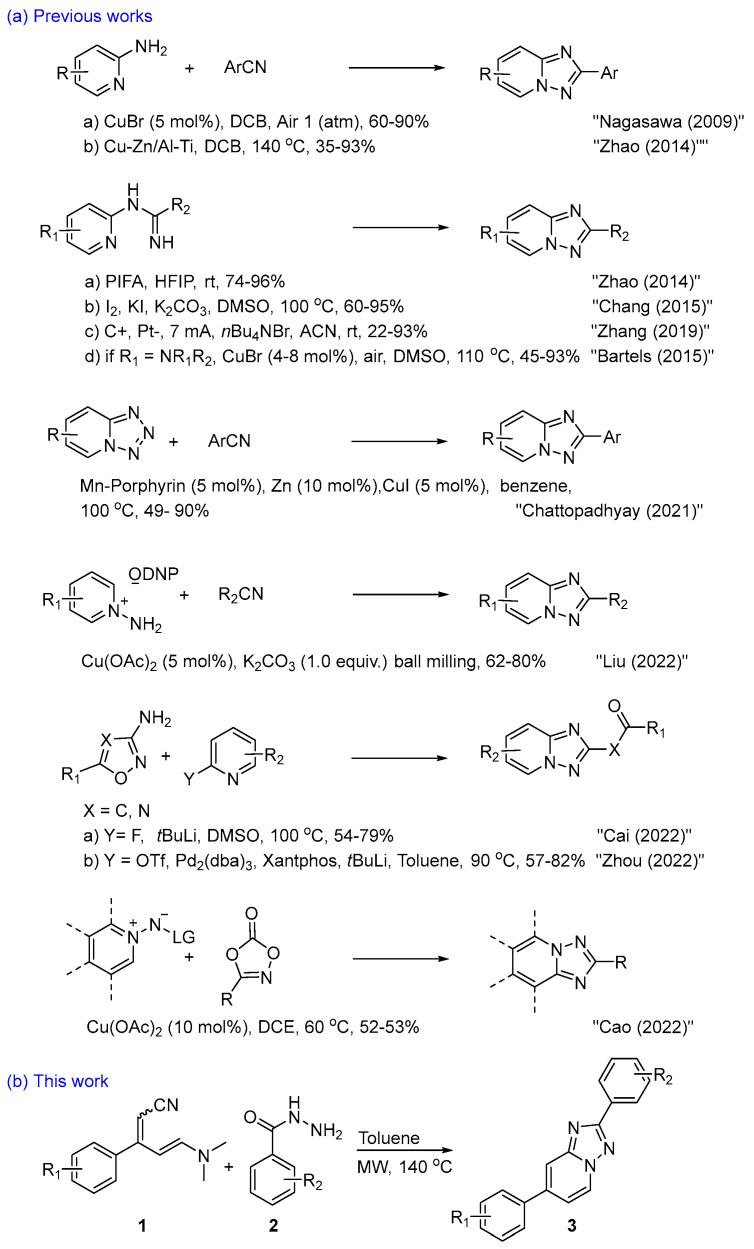
Reported strategies for the synthesis of triazolopyridines and our approach.

Thus, due to the importance of these compounds, a number of methods have been developed for constructing this useful framework (Scheme 1). In 2009, the Nagasawa group first disclosed a facile synthesis of 1,2,4-triazolo[1,5-*a*]pyridines from 2-amino pyridine with nitrile in the presence of CuBr via consecutive addition–oxidative cyclization [15]. Subsequently, numerous research groups have achieved the target compound either by using a transition metal catalyst or through the utilization of external oxidants at the stoichiometric level, such as NaClO/base [16], Pb(OAc)_4_ [17], and MnO_2_ [18]. In 2014, Zhao et al. reported the formation of 1,2,4-triazolo[1,5-*a*]pyridines through the reaction between 2-aminopyridine and nitriles in the presence of Cu-Zn/Al-Ti, a heterogeneous catalyst system under atmospheric air [19].

Zhao et al. described the PIFA-mediated intramolecular annulation of *N*-(2-pyridyl)amidine by forming an oxidative N-N bond [20]. In 2015, Chang and coworkers reported the same reaction in an I_2_/KI reagent system [21]. On the other hand, Zhang and colleagues reported the same intramolecular annulation under metal- and additive-free electrolytic conditions, utilizing *n*Bu_4_Br as both a redox mediator and electrolyte [22]. Bartels et al. developed a CuBr-catalyzed synthesis of 2-amino-triazolopyridine from quanidylpyridines through oxidative cyclization under an air atmosphere [23]. In 2021, Chattopadhyay et al. reported the annulation between 1,2,3,4-tetrazole and nitriles in the presence of a Mn–Porphyrin catalytic system [24]. In 2022, Liu and coworkers developed a mechanochemical method to obtain 1,2,4-triazolo[1,5-*a*]pyridines by the reaction between azinium-*N*-imines and nitriles via a [3 + 2] cycloaddition reaction in the presence of copper acetate [25]. Zhuo et al. developed the reaction from 1,2,4-oxadiazol-3-amines and 2-flouropyidines in a basic medium via tandem S_N_Ar/Boulton–Katritzky rearrangement [26]. Zhuo’s group further developed a palladium-catalyzed tandem reaction between 1,2,4-oxadiazol-3-amines and 2-pyridyl triflate. This cascade reaction proceeds via C-N coupling, followed by Boulton–Kartritzky rearrangement [27]. In 2022, Cao and coworkers reported the reaction of *N*-iminoisoquinolinium ylides with dioxazolone as an acyl nitrile source in the presence of copper acetate, providing 1,2,4-triazolo[1,5-*a*]pyridines via C-H amidation and cyclization [28], and so on [29,30,31,32,33,34,35,36,37,38,39,40,41,42,43,44,45,46,47,48]. Despite the efficiency of these methods in several aspects, there are still some limitations, including the use of solvent in large quantities, longer reaction times, multistep processes, and, in several cases, the requirement for either metal or external catalysts, which will come out as chemical hazards. To overcome these issues, it is crucial to develop more sustainable methods.

In the past two decades, microwave chemistry has attracted significant attention in synthetic organic chemistry due to its rapidity, reproducibility, and efficiency in shorter timeframes compared to conventional approaches [49,50,51]. Moreover, microwave methods play an important role in reducing unwanted byproducts, eliminating the need for hazardous solvents and mitigating harsh reaction conditions. Considering the green chemistry perspective, these types of reactions are environmentally benign, requiring a minimal amount of solvent, enhancing the reaction rates, and profiting from the cost of the overall process.

The nitrile group is an important precursor capable of being transformed into various molecules via reduction, hydration, hydrolysis, nucleophilic addition, and [3 + 2] cycloaddition, leading to the formation of nitrogen-containing heterocyclic compounds [52,53,54]. Enaminonitrile, in particular, is a highly reactive and versatile intermediate utilized for the synthesis of novel heterocyclic compounds [55]. In the course of our investigation into novel metal-free synthesis in our research group [55,56,57,58,59,60,61], we herein present a microwave-mediated cascade reaction to develop 1,2,4-triazolo[1,5-*a*]pyridine (**3**) through the reaction between enaminonitriles (**1**) and benzohydrazides (**2**), as depicted in Scheme 1. To the best of our knowledge, there is no report available to date on the synthesis of triazolo pyridine without use of the catalysts or additives.

## 2. Results and Discussion

In order to find out the optimal reaction conditions, we used enaminonitrile **1m** (1.0 equiv.) and 4-methoxybenzohydrazide **2a** (2.0 equiv.) as starting materials in toluene, stirring at 120 °C for 24 h. Pleasingly, the expected 1,2,4-triazolo[1,5-*a*]pyridine **3m** was obtained with an 83% yield (Table 1, entry 1). Encouraged by this outcome, and aiming to improve the yield of the reaction, we conducted solvent screening, and the results are summarized in Table 1. Solvents such as THF, DMSO, EtOH, and MeOH did not afford the expected product, while a lower yield was observed in the case of DMF and ACN (Table 1, entries 2–4). Notably, pyridine (76%), xylene (69%), and chlorobenzene (79%) delivered **3m** in good yields (Table 1, entries 5–7). To our delight, performing the reaction in dry toluene (86%) and incorporating molecular sieves led to an enhanced yield of 89% under a shorter reaction time of 5 h (Table 1, entries 8, 9). Subsequently, we investigated the impact of varying the equivalence of hydrazide (**2a**). When the equivalence **2a** was reduced from 2.0 equiv. to 1.0 equiv. or 1.5 equiv., the yield of **3a** was significantly dropped to 27% and 51%, respectively (Table 1, entries 10, and 11). We also checked the reaction with different additives; the use of TFA afforded a good yield of 79%, and *p*-TSA resulted in 72% (Table 1, entries 12, and 13). In contrast, the reaction with different bases, including Cs_2_CO_3_, K_2_CO_3_, and KO*t*Bu, failed to afford the product (Table 1, entry 15).

After examining various solvents and additives, we conducted further investigations focusing on the role of reaction temperature. Interestingly, when the reaction was performed in a microwave medium at 140 °C, the desired product **3m** was obtained with an 89% yield within 3 h (Table 1, entry 16). However, we observed that reducing the temperature from 140 °C resulted in a slightly lower yield at both 100 °C and 120 °C (Table 1, entries 17–18). On the other hand, by increasing the reaction temperature to 160 °C and 180 °C, the reactions were completed in 90 min and 40 min, affording **3m** with yields of 81% and 76%, respectively (Table 1, entries 18 and 19). Finally, upon switching to a green solvent such as TBME, a 69% yield of **3m** was obtained. Based on the above screening, we have determined that the optimal reaction conditions for this tandem reaction involve using **1** (1.0 equiv.) and **2** (2.0 equiv.) in dry toluene (1.5 mL) under microwave conditions at 140 °C.

With the optimized conditions in hand, we explored the substrate scope of this methodology. Initially, numerous benzohydrazides were reacted with 4-methoxy enaminonitrile under the standard reaction conditions, and the outcomes are summarized in Table 2. Unsubstituted benzohydrazide worked well under this condition, delivering the anticipated product **3a** in an 83% yield. EDGs on the benzohydrazide, such as methoxy and methyl-substituted derivatives, were well tolerated, affording the desired triazolopyridines in high yields of **3b**-89% and **3c**-82%. Substrates containing strong EWG were then examined, and the CF_3_ group was well tolerated in our optimized conditions, yielding **3d** in 73%. However, the NO_2_ group delivered the equivalent product **3e** in only 24% yield.

Halogen-containing substrates also efficiently underwent this tandem reaction to deliver the expected products in moderate yields (**3f**, 41%, **3g**, 43%). Similar to the aromatic rings, heteroaromatic compounds such as 3-nicotinic hydrazide (76% (**3h**)), 2-thiophenecarboxylic acid hydrazide (94% (**3i**)), and 2-furoic hydrazide (73% (**3j**)) gave the final products in good-to-excellent yields. Additionally, aliphatic acyl hydrazides are compatible under these reactions and displayed good yields (**3k**, 67%, and **3l**, 46%). Along with optimized microwave reaction conditions, we also carried out the reflux reaction at 120 °C with 100 mg of 3Å MS; almost all the obtained products showed similar yields with microwave conditions but with a slightly prolonged reaction time (details are given in Table 2, in the parenthesis).

Encouraged by these outcomes, we next investigated our standard conditions with various enaminonitriles. Almost all the enaminonitriles successfully delivered the final product in very high yield (Table 3). Enaminonitriles with EDGs such as propyl (80%, **3n**) and thiomethyl (71%, **3o**) delivered the products in higher yield. EWGs such as nitro groups delivered the corresponding product **3p** in a slightly moderate yield of 48%. Enaminonitrile with halogens delivered the equivalent products in excellent yields (**3q**, 91%; **3r**, 94%; **3s**, 90%; **3t**, 71%; and **3u**, 67%). These products have the potential to undergo various expansions through coupling reactions with the halogen functional groups. Similar to Table 2, here also, reflux reactions were conducted for some substrates (at 120 °C with 100 mg of 3Å MS, data are in parenthesis of Table 3), which produced the corresponding products in comparable yields with microwave reaction conditions but required a little extra time to complete.

**Scheme 2:**
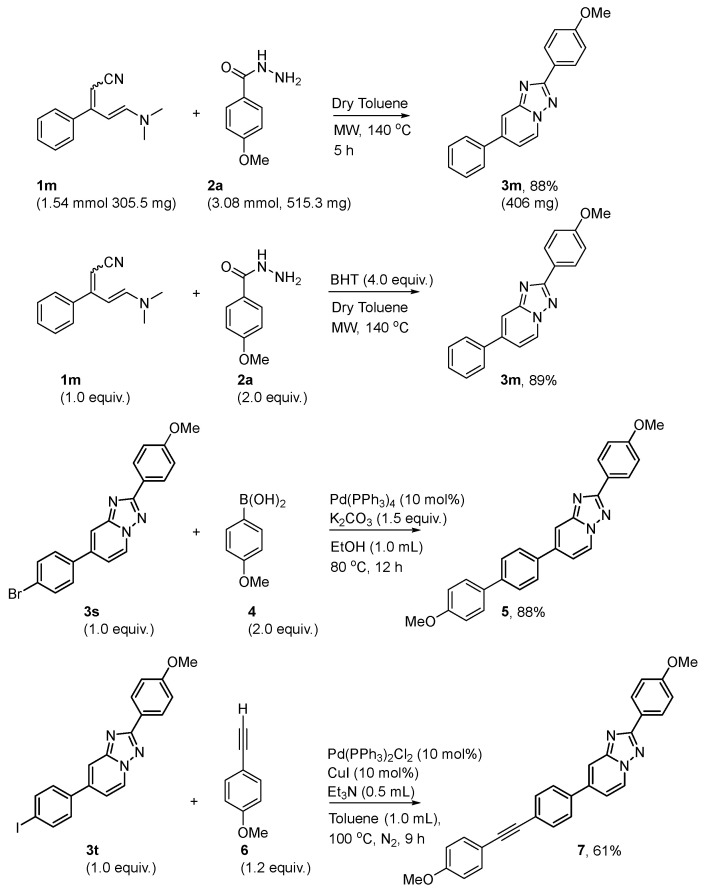
Scale-up and derivatization.

To further expand the synthetic utility of this method, we conducted a scale-up reaction (Scheme 2). An amount of 1.54 mmol of **1m** efficiently reacted with 2.0 equiv. of **2a** to afford the corresponding product **3m** in 88%. We performed a control experiment to gain a deeper insight into the reaction pathways. In the presence of the radical scavenger BHT, **3m** was obtained in an excellent yield of 89%, indicating that this reaction did not go through a radical pathway. From the application point of view, we explored some coupling reactions [62]. Initially, a Suzuki–Miyaura cross-coupling reaction was performed. A triazolopyridine derivative with a bromo functional group (**3s**) reacted with 4-methoxyphenylboronic acid (**4**) in the presence of Pd(PPh_3_)_4_ to afford the coupling product **5** in an 88% yield. Later, we carried out a Sonogashira coupling reaction between the iodo compound (**3t**) and 4-ethylnylanisole (**6**), resulting in the coupling product **7** in a 61% yield.

**Scheme 3:**
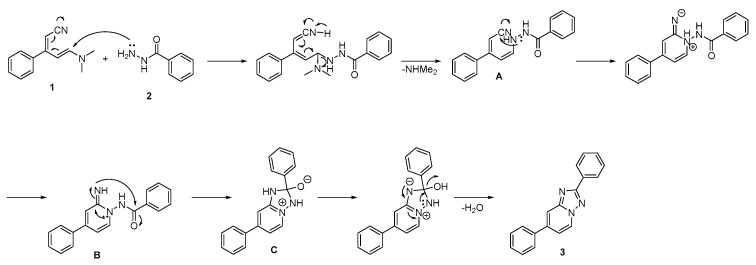
Plausible mechanism of the synthesis of 1,2,4-triazolo[1,5-*a*]pyridine.

Based on the above results, we propose a reaction pathway to explain the possible formation of product **3** (Scheme 3). Initially, compound **1** undergoes transamidation with **2** to deliver the intermediate **A** by the removal of dimethyl amine. The nitrogen lone pair attacks the nitrile moiety of **A**, affording the intermediate **B**, which subsequently undergoes condensation with the carbonyl group to provide intermediate **C**. Finally, the elimination of water leads to the formation of the 1,2,4-triazolo[1,5-*a*]pyridine **3** product.

## 3. Materials and Methods

### 3.1. General Information

Unless noted otherwise, all reagents were purchased from commercial sources and used as received. Reaction progress was monitored using thin-layer chromatography (TLC) using silica gel F_254_ plates. Products were purified using flash column chromatography using silica gel 60 (70–230 mesh) or by using the Biotage ‘Isolera One’ system with indicated solvents. NMR spectra were recorded on a Jeol RESONANCE ECZ 400S (400 MHz for ^1^H NMR and 100 MHz for ^13^C NMR). Chemical shifts are reported in ppm from tetramethylsilane (TMS) with the solvent resonance resulting from incomplete deuteration as the internal reference (CDCl_3_: 7.26 ppm, DMSO-*d_6_*: 2.5 ppm, 3.33 ppm of water peak) or relative to TMS (*δ* 0.0). Data are reported as follows: chemical shift *δ*, multiplicity (s = singlet, d = doublet, t = triplet, q = quartet, m = multiplet, dd = doublet of doublet, td = triplet of doublet, ddd = doublet of doublets of doublets, ddt = doublet of doublet of triplets), coupling constants (Hz), number of protons. High-resolution mass spectra (HRMS) were recorded on either Bruker BioSciences maXis 4G or Thermo Vanquish uhplc system. Melting points were recorded in a Stuart Cole-Parmer SMP30 apparatus. All the microwave reactions were conducted in a Biotage initiator+. The parameters were at 140 °C (pressure 0 bar, power 145–160 W), at 160 °C (pressure 1 bar, power 180–200 W), at 180 °C (pressure 2 bar, power 250–265 W), and at 140 °C in TBME (pressure 6 bar, power 175–190 W).

### 3.2. Typical Procedure for the Preparation of Enaminonitriles [55]

The synthesis of enaminonitriles consists of two steps. The general procedure for step 1, Horner–Wadsworth–Emmons (HWE) reaction with acetophenone: In a 50 mL oven-dried round-bottom flask, sodium hydride (60%, 9.1 mmol, 2.2 equiv.) was added, and the flask was evacuated and backfilled with nitrogen three times. Subsequently, dry tetrahydrofuran (0.5 M) was added, and the mixture was cooled to 0 °C. Diethyl cyanomethylphosphonate (9.1 mmol, 2.2 equiv.) was then slowly added to the reaction mixture and allowed to stir for 30 min. The ice bath was removed, and acetophenone (4.15 mmol, 1.0 equiv.) was added to the reaction mixture, which was then stirred at room temperature. Once the reaction was completed, as monitored using TLC, solvents were evaporated, and the residue was diluted with 30 mL of water. The reaction mixture was extracted three times (20 mL × 3) with ethyl acetate (EtOAc), and the combined organic layers were washed with a sodium chloride (NaCl) solution and dried with Na_2_SO_4_. The solvent was evaporated to obtain the crude product. Purification was carried out using silica gel chromatography using a hexanes/ethyl acetate (9:1) mixture to yield substituted *α,β*-unsaturated 3-phenylbut-2-enenitrile in an *E/Z* mixture (92% yield, 546 mg).

General procedure for step 2, synthesis of enaminonitrile: In a 50 mL oven-dried round-bottom flask, *α,β*-unsaturated 3-phenylbut-2-enenitrile (0.69 mmol, 1.0 equiv) and NH_4_OAc (0.84 mmol, 1.2 equiv.) in DMSO (0.69 mL) were combined, followed by the addition of DMF-DMA (3.49 mmol, 5.0 equiv.). The resulting reaction mixture was stirred at 120 °C until completion, as monitored using TLC. Subsequently, the reaction mixture was allowed to cool to room temperature, diluted with water, and extracted with chloroform (20 mL × 3). The combined organic layers were washed with a saturated NaCl solution, dried over anhydrous Na_2_SO_4_, and then evaporated. The crude product was purified using silica gel chromatography using a hexanes/ethyl acetate mixture (9:1 to 8:2) to yield the (4*E*)-5-(dimethylamino)-3-phenylpenta-2,4-dienenitrile (86%, 118 mg).

### 3.3. Typical Procedure for the Preparation of 1,2,4-triazolo[1,5-a]Pyridines in Microwave Conditions

In an oven-dried microwave vial (0.5–2.0 mL), enaminonitriles (**1**, 0.175 mmol, 1.0 equiv.) and benzohydrazides (**2**, 0.35 mmol, 2.0 equiv.) were added. After evacuation and backfilling with nitrogen three times, dry toluene 1.5 mL was added. The reaction vial was then closed and microwave heating was performed at 140 °C. Once the reaction was completed, as indicated by TLC, the reaction mixture was cooled to room temperature and directly purified using silica gel column chromatography using chloroform/ethyl acetate 10:1 as the eluent.

### 3.4. Typical Procedure for the Preparation of 1,2,4-triazolo[1,5-a]Pyridines in Reflux Conditions

In an oven-dried 25 mL round-bottom flask, enaminonitriles (**1**, 0.175 mmol, 1.0 equiv.), benzohydrazides (**2**, 0.35 mmol, 2.0 equiv.), and 100 mg of 3Å MS were added. After evacuation and backfilling with nitrogen three times, dry toluene 2.0 mL was added. Then, Dean–Stark apparatus was fixed and refluxed at 120 °C. Once the reaction was completed, as monitored using TLC, the reaction mixture was cooled to room temperature and directly purified using silica gel column chromatography using chloroform/ethyl acetate 10:1 as the eluent.

### 3.5. Procedure for the Scale-Up Reaction

In an oven-dried microwave vial (2.0–5.0 mL), enaminonitrile (**1m**, 1.54 mmol, 1.0 equiv.) and 4-methoxybenzohydrazide (**2a**, 3.08 mmol, 2.0 equiv.) were added. After evacuation and backfilling with nitrogen three times, 4.0 mL of dry toluene was added. The reaction vial was then closed and microwave heating was performed at 140 °C. Once the reaction was completed, as monitored using TLC, the reaction mixture was cooled to room temperature and directly purified using silica gel column chromatography using chloroform/ethyl acetate 10:1 to afford the **3m** in an 88% yield (406 mg).

### 3.6. Procedure for the Reaction with Radical Scavenger

In an oven-dried microwave vial (0.5–2.0 mL), enaminonitrile (**1m**, 0.225 mmol, 1.0 equiv.), benzohydrazide (**2a**, 0.45 mmol, 2.0 equiv.), and BHT (0.09 mmol, 4.0 equiv.) were added. After evacuation and backfilling with nitrogen three times, 2.0 mL of dry toluene was added. The reaction vial was then closed, and microwave heating was performed at 140 °C. Once the reaction was completed, as monitored using TLC, the reaction mixture was cooled to room temperature and directly purified using silica gel column chromatography using chloroform/ethyl acetate (10:1) to afford the **3m** in an 89% (60 mg).

### 3.7. Procedure for the Suzuki–Miyaura Coupling Reaction

A reaction mixture of 7-(4-bromophenyl)-2-(4-methoxyphenyl)-[1,2,4]triazolo[1,5-*a*]pyridine (**3s**, 18.1 mg, 0.0476 mmol, 1.0 equiv.), 4-methoxyphenylboronic acid (**4**, 14.5 mg, 2.0 equiv.), Pd(PPh_3_)_4_ (5.5 mg, 0.1 equiv.), and K_2_CO_3_ (9.9 mg, 1.5 equiv.) in degassed ethanol (1.0 mL) was stirred at 80 °C for 12 h using sand bath. The reaction progress was monitored using TLC, and once the reaction was completed, the reaction mixture was concentrated under reduced pressure, diluted with ethyl acetate (10 mL), and washed with brine (5 mL × 2). The aqueous layer was extracted with DCM (10 mL × 2). The combined organic layers were dried over MgSO_4_ and concentrated under reduced pressure to give the crude product, which was purified using silica gel column chromatography using DCM: EtOAc (0 -> 5%) to afford the coupled product **5** in an 88% yield (17.1 mg) as a pale-yellow solid.

### 3.8. Procedure for the Sonogashira Coupling Reaction

A mixture of 7-(4-iodophenyl)-2-(4-methoxyphenyl)-[1,2,4]triazolo[1,5-*a*]pyridine (**3t**, 16.1 mg, 0.0377 mmol, 1.0 equiv), 4-ethynylanisole (**6**, 6.0 mg, 1.2 equiv.), Pd(PPh_3_)_2_Cl_2_ (2.6 mg, 0.1 equiv.), CuI (0.7 mg, 0.1 equiv.), and Et_3_N (0.5 mL) in toluene (1.0 mL) was stirred at 100 °C for 9 h under nitrogen atmosphere. The reaction progress was monitored using TLC, and once the reaction was completed, the reaction mixture was concentrated under reduced pressure, diluted with ethyl acetate (10mL), and washed with brine (5 mL × 2). The water layer was extracted with dichloromethane (10 mL × 2). The combined organic layers were dried over MgSO_4_ and concentrated under reduced pressure to give the crude product, which was purified using silica gel column chromatography by using DCM: EtOAc (0 to 5%) to afford the coupled product **7** in 61% yield (9.9 mg) as a pale-yellow solid.

### 3.9. Anlytical Data of Synthezised Compounds

7-(4-methoxyphenyl)-2-phenyl-[1,2,4]triazolo[1,5-*a*]pyridine **(3a)**

M.W: yield 82.6% (43.0 mg, SM was used 39.5 mg), reflux: yield 85.1% (39.2 mg, SM was used 39.5 mg); pale yellow solid; mp: 141–143 °C; ^1^H NMR (400 MHz, CDCl_3_): *δ* 8.59 (dd, *J* = 4.6, 2.4 Hz, 1H), 8.32–8.29 (m, 2H), 7.89 (dd, *J* = 3.2, 0.8 Hz, 1H), 7.65–7.60 (m, 2H), 7.54–7.47 (m, 3H), 7.25 (td, *J* = 7.9, 2.0 Hz, 1H), 7.04 (dd, *J* = 5.6, 3.4 Hz, 2H), 3.88 (s, 3H); ^13^C NMR (100 MHz, CDCl_3_): *δ* 160.7, 160.7, 152.0, 130.4, 130.2, 128.9, 128.4, 128.1, 127.5, 114.8, 113.7, 113.6, 112.0, 111.9, 55.6; HRMS (ESI) m/z [M + H]^+^: calcd for C_19_H_16_N_3_O: 302.1293; Found: 302.1289.

2,7-bis(4-methoxyphenyl)-[1,2,4]triazolo[1,5-*a*]pyridine **(3b)**

M.W: yield 88.3% (51.2 mg, SM was used 40.0 mg), reflux: yield 88.3% (67.5 mg, SM was used 52.7 mg); pale yellow solid; mp: 188–190 °C; *^1^*H NMR (400 MHz, CDCl_3_): *δ* 8.56 (d, *J* = 7.2 Hz, 1H), 8.25 (d, *J* = 8.8 Hz, 2H), 7.88 (s, 1H), 7.63 (d, *J* = 8.8 Hz, 2H), 7.24 (d, *J* = 6.8 Hz, 1H), 7.05–7.01 (m, 4H), 3.88 (s, 3H), 3.88 (S, 3H); ^13^C NMR (100 MHz, CDCl_3_): *δ* 164.0, 161.6, 160.7, 151.7, 143.1, 130.2, 129.0, 128.4, 128.0, 122.9, 114.8, 114.3, 113.5, 111.6, 55.6, 55.5; HRMS (ESI) m/z [M + H]^+^: calcd for C_20_H_18_N_3_O_2_: 332.1399; Found: 332.1393.

7-(4-methoxyphenyl)-2-(p-tolyl)-[1,2,4]triazolo[1,5-*a*]pyridine **(3c)**

Yield 81.3% (48.9 mg, SM was used 43.5 mg); yellow solid; mp: 155–157 °C; *^1^*H NMR (400 MHz, CDCl_3_): *δ* 8.55 (d, *J* = 6.8 Hz, 1H), 8.18 (d, *J* = 7.6 Hz, 2H), 7.84 (s, 1H), 7.60 (d, *J* = 8.4 Hz, 2H), 7.02 (d, *J* = 8.4 Hz, 2H), 7.19 (dd, *J* = 5.6, 1.6 Hz, 1H), 7.01 (d, *J* = 8.4 Hz, 2H), 3.86 (S, 3H), 2.41 (s, 3H); ^13^C NMR (100 MHz, CDCl_3_): *δ* 164.7, 160.5, 152.1, 142.6, 140.4, 130.3, 129.6, 128.3, 128.0, 127.9, 127.3, 114.8, 113.3, 111.9, 55.5, 21.6; HRMS (ESI) m/z [M + H]^+^: calcd for C_20_H_18_N_3_O: 316.1449; Found: 316.1445.

7-(4-methoxyphenyl)-2-(4-(trifluoromethyl)phenyl)-[1,2,4]triazolo[1,5-*a*]pyridine **(3d)**

Yield 73.2% (60.3 mg, SM was used 50.8 mg); pale yellow solid; mp: 219–221 °C; *^1^*H NMR (400 MHz, CDCl_3_): *δ* 8.61 (d, *J* = 7.2 Hz, 1H), 8.43 (d *J* = 7.6 Hz, 2H), 7.90 (s, 1H), 7.77 (d, *J* = 7.6 Hz, 2H), 7.64 (d, *J* = 8.0 Hz, 2H), 7.30 (d, *J* = 7.2 Hz, 1H), 7.05 (d, *J* = 8.4 Hz, 2H), 3.89 (s, 3H); ^13^C NMR (100 MHz, CDCl_3_): *δ* 161.7, 152.0, 143.3, 139.4, 135.3, 130.1, 129.1, 128.0, 127.1, 123.4, 119.5, 114.4, 113.5, 112.4, 55.5, 21.3. ^19^F NMR (376 MHz, CDCl_3_): *δ* -62.7; HRMS (ESI) m/z [M + H]^+^: calcd for C_20_H_15_F_3_N_3_O: 370.1167; Found: 370.1165.

7-(4-methoxyphenyl)-2-(4-nitrophenyl)-[1,2,4]triazolo[1,5-*a*]pyridine **(3e)**

M.W: yield 24.0% (5.6 mg, SM was used 15.3 mg), reflux: yield 23.8% (8.6 mg, SM was used 24.0 mg); pale yellow solid; mp: 177–179 °C; *^1^*H NMR (400 MHz, CDCl_3_): *δ* 8.62 (d, *J* = 7.2 Hz, 1H), 8.48 (d, *J* = 8.8 Hz, 2H), 8.36 (d, *J* = 8.8 Hz, 2H), 7.91 (s, 1H), 7.65 (d, *J* = 8.8 Hz, 2H), 7.33 (dd, *J* = 5.6, 1.6 Hz, 1H), 7.06 (d, *J* = 8.8 Hz, 2H), 3.89 (s, 3H); ^13^C NMR (100 MHz, DMSO-*d*_6_): *δ* 161.4, 160.0, 151.6, 148.2, 142.0, 136.4, 128.4, 127.9, 127.5, 123.6, 114.4, 113.7, 111.1, 54.9; HRMS (ESI) m/z [M + H]^+^: calcd for C_20_H_15_N_4_O_3_: 347.1144; Found: 347.1135.

2-(4-chlorophenyl)-7-(4-methoxyphenyl)-[1,2,4]triazolo[1,5-*a*]pyridine **(3f)**

M.W: yield 40.8% (28.8 mg, SM was used 48.2 mg), reflux: yield 42.1% (14.5, SM was used 23.5 mg); pale yellow solid; mp: 202–204 °C; *^1^*H NMR (400 MHz, CDCl_3_): *δ* 8.57 (d, *J* = 6.4 Hz, 1H), 8.23 (dt, *J* = 8.8, 2.2 Hz, 2H), 7.86 (q, *J* = 0.9 Hz, 1H), 7.62 (dt, *J* = 9.2, 2.6 Hz, 2H), 7.47 (dt, *J* = 8.8, 2.2 Hz, 2H), 7.26 (dd, *J* = 4.4, 2.4 Hz, 1H), 7.04 (dt, *J* = 8.8, 2.7 Hz, 2H), 3.88 (s, 3H); ^13^C NMR (100 MHz, CDCl_3_): *δ* 163.5, 160.7, 152.1, 143.1, 136.4, 130.1, 129.2, 129.2, 128.8, 128.4, 128.0, 114.9, 113.8, 112.0, 55.6; HRMS (ESI) m/z [M + H]^+^: calcd for C_19_H_15_ClN_3_O: 336.0904; Found: 336.0899.

2-(4-bromophenyl)-7-(4-methoxyphenyl)-[1,2,4]triazolo[1,5-*a*]pyridine **(3g)**

Yield 43.0% (31.3 mg, SM was used 43.8 mg); pale yellow solid; mp: 209–211 °C; *^1^*H NMR (400 MHz, CDCl_3_): *δ* 8.60 (d, *J* = 7.2 Hz, 1H), 8.26 (d, *J* = 9.2 Hz, 2H), 7.91 (s, 1H), 7.65 (d, *J* = 8.4 Hz, 2H), 7.54 (d, *J* = 8.8 Hz, 2H), 7.22–7.20 (m, 1H), 7.03 (d, *J* = 8.4 Hz, 2H), 3.88 (s, 3H); ^13^C NMR (100 MHz, DMSO-*d*_6_): *δ* 163.5, 160.6, 151.2, 140.2, 136.2, 131.5, 128.7, 128.3, 128.0, 123.0, 122.0, 114.0, 112.3, 111.7, 54.9; HRMS (ESI) m/z [M + H]^+^: calcd for C_19_H_15_BrN_3_O: 380.0398; Found: 380.0390.

7-(4-methoxyphenyl)-2-(pyridin-3-yl)-[1,2,4]triazolo[1,5-*a*]pyridine **(3h)**

Yield 76.3% (48.4 mg, SM was used 47.8 mg); pale yellow solid; mp: 173–175 °C; *^1^*H NMR (400 MHz, CDCl_3_): *δ* 9.53 (s, 1H), 8.71 (d, *J* = 5.2 Hz, 1H), 8.61–8.58 (m, 2H), 7.88 (q, *J* = 0.9 Hz, 1H), 7.64 (dt, *J* = 8.8, 2.7 Hz, 2H), 7.48 (dd, *J* = 5.2, 5.0 Hz, 2H), 7.29 (dd, *J* = 5.6, 1.8 Hz, 1H), 7.05 (dt, *J* = 9.2, 2.5 Hz, 2H), 3.88 (s, 3H); ^13^C NMR (100 MHz, CDCl_3_): *δ* 162.2, 160.7, 152.4, 150.3, 148.2, 143.1, 135.2, 130.1, 128.4, 128.1, 127.4, 124.0, 114.9, 114.0, 112.2, 55.6; HRMS (ESI) m/z [M + H]^+^: calcd for C_18_H_15_N_4_O: 303.1245; Found: 303.1242.

7-(4-methoxyphenyl)-2-(thiophen-2-yl)-[1,2,4]triazolo[1,5-*a*]pyridine **(3i)**

M.W: yield 94.1% (57.2 mg, SM was used 45.1 mg), reflux: yield 85.2% (28.0 mg, SM was used 24.4 mg); pale yellow solid; mp: 150–152 °C; *^1^*H NMR (400 MHz, CDCl_3_): *δ* 8.54 (d, *J* = 6.8 Hz, 1H), 7.90 (dd, *J* = 2.8, 0.8 Hz,1H), 7.82 (d, *J* = 0.8 Hz, 1H), 7.60 (dt, *J* = 8.4, 2.6 Hz 2H), 7.45 (dd, *J* = 3.6, 1.4 Hz, 1H), 7.22 (dd, *J* = 5.2, 1.8 Hz, 1H), 7.17 (dd, *J* = 3.6, 1.6 Hz, 1H), 7.02 (dt, *J* = 9.2, 2.4 Hz, 2H), 3.86 (s, 3H); ^13^C NMR (100 MHz, CDCl_3_): *δ* 160.6, 152.0, 143.0, 133.6, 130.1, 128.4, 128.2, 128.0, 127.9, 127.8, 114.8, 113.5, 111.7, 55.5; HRMS (ESI) m/z [M + H]^+^: calcd for C_17_H_14_N_3_OS: 308.0857; Found: 308.0852.

2-(furan-2-yl)-7-(4-methoxyphenyl)-[1,2,4]triazolo[1,5-*a*]pyridine **(3j)**

Yield 72.5% (46.6 mg, SM was used 50.5 mg); pale yellow solid; mp: 137–139 °C; *^1^*H NMR (400 MHz, CDCl_3_): *δ* 8.59 (d, *J* = 6.8 Hz, 1H), 7.87 (s,1H), 7.64–7.62 (m, 3H), 7.30–7.26 (m, 2H), 7.05 (d, *J* = 6.8 Hz, 2H), 6.60 (s, 1H), 3.88 (s, 3H); ^13^C NMR (100 MHz, CDCl_3_): *δ* 160.7, 157.2, 151.7, 146.1, 144.4, 143.4, 130.1, 128.5, 128.1, 114.8, 113.9, 112.0, 111.9, 111.7, 55.6; HRMS (ESI) m/z [M + H]^+^: calcd for C_17_H_14_N_3_O_2_: 292.1086; Found: 292.1081.

7-(4-methoxyphenyl)-2-methyl-[1,2,4]triazolo[1,5-*a*]pyridine **(3k)**

M.W: yield 66.7% (30.6 mg, SM was used 43.8 mg), reflux: 65.5% (13.8 mg, SM was used 20.0 mg); brown solid; mp: 192–194 °C; *^1^*H NMR (400 MHz, CDCl_3_): *δ* 8.47 (d, *J* = 7.2 Hz, 1H), 7.73 (d, *J* = 0.8 Hz, 1H), 7.58 (dt, *J* = 9.2, 2.5 Hz, 2H), 7.17 (dd, *J* = 5.2, 2.0 Hz, 1H), 7.01 (dt, *J* = 9.2, 2.6 Hz, 2H), 3.86 (s, 3H), 2.60 (s, 3H); ^13^C NMR (100 MHz, CDCl_3_): *δ* 164.4, 160.6, 151.7, 142.6, 130.3, 128.4, 127.6, 114.7, 112.9, 111.5, 55.5, 14.6; HRMS (ESI) m/z [M + H]^+^: calcd for C_14_H_14_N_3_O: 240.1136; Found: 240.1131.

2-heptyl-7-(4-methoxyphenyl)-[1,2,4]triazolo[1,5-*a*]pyridine **(3l)**

Yield 46.2% (31.4 mg, SM was used 48.0 mg); pale yellow solid; mp: 224–226 °C; *^1^*H NMR (400 MHz, CDCl_3_): *δ* 8.47 (d, *J* = 7.2 Hz, 1H), 7.74 (s, 1H), 7.57 (d, *J* = 8.8 Hz, 2H), 7.15 (dd, *J* = 5.6, 1.6 Hz, 1H), 7.00 (d, *J* = 4.4 Hz, 2H), 3.84 (s, 3H), 2.90 (t, *J* = 7.8 Hz, 2H), 1.86 (p, *J* = 5.8 Hz, 2H), 1.41–1.26 (m, 9H), 0.85 (t, *J* = 6.6 Hz, 3H); ^13^C NMR (100 MHz, CDCl_3_): *δ* 168.3, 160.5, 151.7, 142.4, 130.4, 128.3, 127.7, 114.7, 112.8, 111.7, 55.5, 31.8, 29.5, 29.2, 29.0, 28.5, 22.7, 14.2; HRMS (ESI) m/z [M + H]^+^: calcd for C_20_H_26_N_3_O: 324.2075; Found: 324.2070.

2-(4-methoxyphenyl)-7-phenyl-[1,2,4]triazolo[1,5-*a*]pyridine **(3m)**

M.W: yield 89.0% (64.9 mg, SM was used 48.0 mg), reflux: yield 89.0% (39.7 mg, SM was used 29.4 mg); pale yellow solid; mp: 179–181 °C; *^1^*H NMR (400 MHz, CDCl_3_): *δ* 8.60–8.57 (m, 1H), 8.24 (d, *J* = 8.4 Hz, 2H), 7.90 (s, 1H), 7.69–7.61 (m, 2H), 7.55–7.44 (m, 3H), 7.27–7.23 (m, 1H), 7.03 (dd, *J* = 6.8, 2.0 Hz, 2H), 3.88 (s, 3H); ^13^C NMR (100 MHz, CDCl_3_): *δ* 164.6, 161.4, 152.0, 143.0, 138.1, 129.4, 129.1, 128.9, 128.0, 127.2, 123.3, 114.2, 113.4, 112.8, 55.5; HRMS (ESI) m/z [M + H]^+^: calcd for C_19_H_16_N_3_O: 302.1293; Found: 302.1288.

2-(4-methoxyphenyl)-7-(4-propylphenyl)-[1,2,4]triazolo[1,5-*a*]pyridine **(3n)**

M.W: yield 80.0% (48.0 mg, SM was used 42.0 mg), reflux: yield 84.1% (24.8 mg, SM was used 20.7 mg); pale yellow solid; mp: 158–160 °C; *^1^*H NMR (400 MHz, CDCl_3_): *δ* 8.58 (d, *J* = 7.6 Hz, 1H), 8.24 (d, *J* = 8.8 Hz, 2H), 7.89 (d, *J* = 1.2 Hz, 1H), 7.59 (d, *J* = 8.0 Hz, 2H), 7.32 (d, *J* = 8.0 Hz, 2H), 3.88 (s, 3H), 2.66 (t, *J* = 7.6 Hz, 2H), 2.04 (h, *J* = 3.6 Hz, 2H), 0.98 (t, *J* = 7.4 Hz, 3H); ^13^C NMR (100 MHz, CDCl_3_): *δ* 164.5, 161.4, 152.0, 144.1, 143.1, 135.3, 129.5, 128.9, 127.9, 127.0, 123.3, 114.2, 113.4, 112.3, 55.4, 37.8, 24.5, 13.9; HRMS (ESI) m/z [M + H]^+^: calcd for C_22_H_22_N_3_O: 344.1763; Found: 344.1755.

2-(4-methoxyphenyl)-7-(4-(methylthio)phenyl)-[1,2,4]triazolo[1,5-*a*]pyridine **(3o)**

M.W: yield 70.5% (59.0 mg, SM was used 58.8 mg), reflux: yield 54.2% (16.7 mg, SM was used 21.7 mg); pale yellow solid; mp: 201–203 °C; *^1^*H NMR (400 MHz, CDCl_3_): *δ* 8.57 (d, *J* = 7.6 Hz, 1H), 8.23 (d, *J* = 8.0 Hz, 2H), 7.88 (s, 1H), 7.59 (d, *J* = 7.6 Hz, 2H), 7.35 (d, *J* = 8.4 Hz, 2H), 7.23–7.21 (m, 1H), 7.01 (d, *J* = 8.8 Hz, 2H) 3.87 (s, 3H), 2.53 (S, 3H); ^13^C NMR (100 MHz, CDCl_3_): *δ* 164.5, 161.7, 151.9, 142.7, 140.7, 134.6, 129.2, 128.1, 127.5, 127.2, 123.2, 114.4, 113.2, 112.1, 55.5, 15.7; HRMS (ESI) m/z [M + H]^+^: calcd for C_20_H_18_N_3_OS: 348.1170; Found: 348.1168.

2-(4-methoxyphenyl)-7-(2-nitrophenyl)-[1,2,4]triazolo[1,5-*a*]pyridine **(3p)**

M.W: yield 48.2% (27.5, SM was used 40.1 mg), reflux: yield 47.1% (29.0 mg, SM was used 43.3 mg); pale yellow solid; mp: 170–172 °C; *^1^*H NMR (400 MHz, CDCl_3_): *δ* 8.57 (d, *J* = 6.8 Hz, 1H), 8.23 (d, *J* = 9.2 Hz, 2H), 8.04 (d, *J* = 7.2 Hz 1H), 7.71 (td, *J* = 7.6, 1.3 Hz, 1H), 7.68 (s, 1H), 7.61 (td, *J* = 7.6, 1.1 Hz, 1H), 7.50 (dd, *J* = 6.8, 0.8 Hz, 1H), 7.02 (d, *J* = 8.8 Hz, 2H), 6.89 (dd, *J* = 5.6, 1.6 Hz, 1H), 3.88 (S, 3H); ^13^C NMR (100 MHz, CDCl_3_): *δ* 164.7, 161.5, 151.4, 148.5, 140.1, 134.0, 133.4, 131.8, 129.8, 129.0, 127.9, 125.0, 123.0, 114.7, 114.3, 114.2, 55.5; HRMS (ESI) m/z [M + H]^+^: calcd for C_19_H_15_N_4_O_3_: 347.1144; Found: 347.1138.

7-(4-fluorophenyl)-2-(4-methoxyphenyl)-[1,2,4]triazolo[1,5-*a*]pyridine **(3q)**

Yield 91.1% (62.8 mg, SM was used 46.6 mg); pale yellow solid; mp: 197–199 °C; *^1^*H NMR (400 MHz, CDCl_3_): *δ* 8.59 (d, *J* = 6.8 Hz, 1H), 8.24 (d, *J* = 8.8 Hz, 2H), 7.87 (s, 1H), 7.67–7.63 (m, 2H), 7.23–7.18 (m, 3H), 7.03 (d, *J* = 9.2 Hz, 2H), 3.88 (s, 3H); ^13^C NMR (100 MHz, CDCl_3_): *δ* 164.8, 164.4, 162.3, 161.6, 151.7, 142.3, 134.2, 129.1, 129.0, 129.0, 128.2, 123.0, 116.4 (d, *J* = 21.1 Hz), 114.3, 113.4, 112.6, 55.5; ^19^F NMR (376 MHz, CDCl_3_): *δ* -112.7; HRMS (ESI) m/z [M + H]^+^: calcd for C_19_H_15_FN_3_O: 320.1199; Found: 320.1194.

7-(4-chlorophenyl)-2-(4-methoxyphenyl)-[1,2,4]triazolo[1,5-*a*]pyridine **(3r)**

Yield 93.6% (57.4 mg, SM was used 42.5 mg); pale yellow solid; mp: 231–233 °C; *^1^*H NMR (400 MHz, CDCl_3_): *δ* 8.59 (d, *J* = 7.2 Hz, 1H), 8.25 (d, *J* = 8.4 Hz, 2H), 7.89 (s, 1H), 7.60 (d, *J* = 8.4 Hz, 2H), 7.49 (d, *J* = 8.4 Hz, 2H), 7.20 (d, *J* = 6.8 Hz, 1H), 7.03 (d, *J* = 8.8 Hz, 2H), 3.88 (s, 3H); ^13^C NMR (100 MHz, CDCl_3_): *δ* 164.7, 161.8, 151.8, 142.0, 136.6, 135.6, 129.7, 129.2, 128.5, 128.3, 123.2, 114.4, 113.2, 112,9 55.5; HRMS (ESI) m/z [M + H]^+^: calcd for C_19_H_15_ClN_3_O: 336.0903; Found: 336.0898.

7-(4-bromophenyl)-2-(4-methoxyphenyl)-[1,2,4]triazolo[1,5-*a*]pyridine **(3s)**

Yield 90.1% (58.6 mg, SM was used 47.5 mg); pale yellow solid; mp: 220–222 °C; *^1^*H NMR (400 MHz, CDCl_3_): *δ* 8.61 (d, *J* = 6.8 Hz, 1H), 8.23 (dt, *J* = 8.8, 2.6 Hz, 2H), 7.19 (s, 1H), 7.65 (dt, *J* = 8.4, 2.0 Hz, 2H), 7.54 (dt, *J* = 8.4, 2.1 Hz, 2H), 7.19 (dd, *J* = 5.6, 1.8 Hz, 1H), 7.03 (dt, *J* = 8.8, 2.5 Hz, 2H), 3.88 (s, 3H); ^13^C NMR (100 MHz, DMSO-*d*_6_): *δ* 163.5, 160.6, 151.2, 140.2, 136.1, 131.6, 128.6, 128.3, 128.0, 122.9, 122.0, 114.0, 112.3, 111.6, 54.9; HRMS (ESI) m/z [M + H]^+^: calcd for C_19_H_15_BrN_3_O: 380.0398; Found: 380.0393.

7-(4-iodophenyl)-2-(4-methoxyphenyl)-[1,2,4]triazolo[1,5-*a*]pyridine **(3t)**

Yield 70.7% (50.1 mg, SM was used 53.9 mg), reflux: yield 75.6% (28.7 mg, SM was used 28.7 mg); pale yellow solid; mp: 228–230 °C; M.W: *^1^*H NMR (400 MHz, CDCl_3_): *δ* 8.60 (d, *J* = 6.8 Hz, 1H), 8.23 (dt, *J* = 9.2, 2.6 Hz, 2H), 7.88 (s, 1H), 7.85 (d, *J* = 8.8 Hz, 2H), 7.40 (d, *J* = 8.4 Hz, 2H), 7.20 (dd, *J* = 5.2, 1.8 Hz, 1H), 7.03 (dt, *J* = 8.8, 2.4 Hz, 2H), 3.87 (S, 3H); ^13^C NMR (100 MHz, DMSO-*d*_6_): *δ* 163.5, 160.6, 151.1, 140.4, 137.5, 136.5, 128.6, 128.3, 128.0, 123.0, 113.9, 112.2, 111.5, 94.6, 54.9; HRMS (ESI) m/z [M + H]^+^: calcd for C_19_H_15_IN_3_O: 428.0259; Found: 428.0254.

7-(2,5-dichlorophenyl)-2-(4-methoxyphenyl)-[1,2,4]triazolo[1,5-*a*]pyridine **(3u)**

Yield 67% (38.9 mg, SM was used 41.9 mg); pale yellow solid; mp: 225–227 °C; *^1^*H NMR (400 MHz, CDCl_3_): *δ* 8.60 (d, *J* = 7.2 Hz, 1H), 8.25 (d, *J* = 8.4 Hz, 2H), 7.76 (d, *J* = 2.0 Hz, 1H), 7.46 (d, *J* = 8.8 Hz, 1H), 7.42 (d, *J* = 2.8 Hz, 1H), 7.36 (dd, *J* = 8.8 Hz, *J* = 2.8 Hz, 1H), 7.05 (dd, *J* = 7.2 Hz, *J* = 2.0 Hz, 1H), 7.03 (d, *J* = 8.4 Hz, 2H), 3.89 (s, 3H); ^13^C NMR (100 MHz, CDCl_3_): *δ* 165.1, 161.7, 151.5, 139.9, 139.3, 133.4, 131.7, 131.0, 130.9, 130.0, 129.2, 127.7, 123.4, 116.5, 115.2, 114.4, 55.5; HRMS (ESI) m/z [M + H]^+^: calcd for C_19_H_14_Cl_2_N_3_O: 370.0514; Found: 370.0508.

7-(4′-methoxy-[1,1′-biphenyl]-4-yl)-2-(4-methoxyphenyl)-[1,2,4]triazolo[1,5-*a*]pyridine **(5)**

Yield 88% (17.1 mg, SM used 18.1 mg); pale yellow solid; mp: 208–210 °C; NMR was conducted at 373 K because of poor solubility of **5.** *^1^*H NMR (400 MHz, DMSO-*d*_6_): *δ* 8.91 (d, *J* = 7.6 Hz, 1H), 8.17 (d, *J* = 8.8 Hz, 2H), 8.08 (d, *J* = 1.2 Hz, 1H), 7.93 (d, *J* = 8.0 Hz, 2H), 7.78 (d, *J* = 8.0 Hz, 2H), 7.69 (d, *J* = 8.8 Hz, 2H), 7.51 (dd, *J* = 7.6 Hz, *J* = 1.2 Hz, 1H), 7.10 (d, *J* = 8.8 Hz, 2H), 7.07 (d, *J* = 8.8 Hz, 2H), 3.87 (s, 3H), 3.84 (s, 3H); *^13^*C NMR (100 MHz, DMSO-*d*_6_) *δ* 163.4, 160.5, 159.0, 151.2, 141.0, 140.0, 134.9, 131.3, 128.1, 127.9, 127.2, 126.9, 126.3, 123.0, 114.2, 113.9, 112.3, 111.1, 54.9, 54.8; HRMS (ESI) m/z [M + H]^+^: calcd for C_26_H_22_N_3_O_2_: 408.1712; Found: 408.1707.

2-(4-methoxyphenyl)-7-(4-((4-methoxyphenyl)ethynyl)phenyl)-[1,2,4]triazolo[1,5-*a*]pyridine **(7)**

Yield 61% (9.9 mg, SM used 16.1 mg); pale yellow solid; mp: 233–235 °C; NMR was conducted at 413 K because of poor solubility of **7**. *^1^*H NMR (400 MHz, DMSO-*d*_6_) *δ* 8.92 (d, *J* = 7.6 Hz, 1H), 8.17 (d, *J* = 8.8 Hz, 2H), 8.09 (d, *J* = 1.6 Hz, 1H), 7.91 (d, *J* = 8.0 Hz, 2H), 7.67 (d, *J* = 8.0 Hz, 2H), 7.52 (d, *J* = 8.8 Hz, 2H), 7.50 (dd, *J* = 7.6 Hz, *J* = 1.6 Hz, 1H), 7.10 (d, *J* = 8.8 Hz, 2H), 7.01 (d, *J* = 8.8 Hz, 2H), 3.87 (s, 3H), 3.83 (s, 3H); *^13^*C NMR (100 MHz, DMSO-*d*_6_) *δ* 163.4, 160.5, 159.3, 151.0, 140.3, 136.3, 132.2, 131.1, 127.9, 127.8, 126.4, 123.0, 122.9, 114.0, 113.9, 113.8, 112.0, 111.4, 90.4, 87.0, 54.8 (two methoxy groups appeared at one peak); HRMS (ESI) m/z [M + H]^+^: calcd for C_28_H_22_N_3_O_2_: 432.1712; Found: 432.1707.

## 4. Conclusions

We demonstrated a microwave-mediated cascade synthesis of 1,2,4-triazolo [1,5-*a*]pyridines in good-to-excellent yields. Various enaminonitriles and benzo- and hetero- hydrazides successfully yielded the final products. Additionally, our protocol can be used for scale-up synthesis, and facile transformation of the resulting triazolopyridines was explored. This strategy operates without the need for catalysts, additives, or workups and utilizes a minimal amount of solvent. These features make this methodology highly attractive.

## Figures and Tables

**Table 1:**
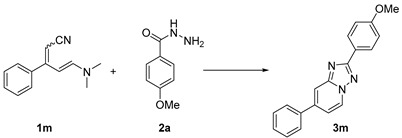
Optimization of reaction conditions ^a^.

Entry	Solvent	Additive	Time (h)	Yield (%) ^b^
1	Toluene		24	83
2	Solvents ^c^		24	NR ^d^
3	DMF		24	20
4	ACN		24	17
5	Pyridine		24	76
6	Xylene		24	69
7	Chlorobenzene		24	79
8	Dry Toluene		12	86
9 ^e^	Dry Toluene	3Å MS	5	89
10 ^f^	Dry Toluene		13	27
11 ^g^	Dry Toluene		26	51
12 ^h^	Dry Toluene	TFA	2	79
13 ^h^	Dry Toluene	*p*-TSA	1	72
14 ^h^	Dry Toluene	MsOH	0.5	50
15 ^h^	Dry Toluene	Bases ^i^	2	NR
16 ^j,k^	Dry Toluene		3	89
17 ^l,k^	Dry Toluene		6	28
18 ^m,k^	Dry toluene		6	62
19 ^n,k^	Dry Toluene		90 min	81
20 ^o,k^	Dry Toluene		40 min	76
21 ^p,k^	TBME		3	69

^a^ Unless otherwise mentioned, all reactions were performed in **1m** (0.10 mmol, 1.0 equiv.), **2a** (0.20 mmol, 2.0 equiv.), and solvent (2.0 mL), stirred at 120 °C under conventional heating for the indicated time. ^b^ Isolated yield. ^c^ Solvents: THF, DMSO, EtOH, MeOH. ^d^ NR—No reaction. ^e^ An amount of 100 mg of 3Å MS was used. ^f^ Used 1.0 equiv. of **2a**. ^g^ Used 1.5 equiv. of **2a**. ^h^ An amount of 1.5 equiv. of additive was used and stirred at 140 °C. ^i^ Bases: Cs_2_CO_3_, K_2_CO_3_, and KO*t*Bu. ^j^ Conducted under microwave conditions at 140 °C (pressure 0 bar, power 145–160 W). ^k^ An amount of 1.5 mL of solvent was used. ^l^ Conducted under microwave conditions at 100 °C (pressure 1 bar, power 92–105 W). ^m^ Conducted under microwave conditions at 120 °C (pressure 0 bar, power 105–115 W). ^n^ Conducted under microwave conditions at 160 °C (pressure 1 bar, power 180–200 W). ^o^ Conducted under microwave conditions at 180 °C (pressure 2 bar, power 250–265 W). ^p^ Conducted under microwave conditions at 140 °C (pressure 6 bar, power 175–190 W).

**Table 2:**
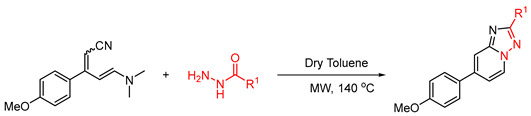
Substrate scope of acylhydrazides ^a^.

Entry No	Compound Code	R^1^	Time (h)	Yield (%)	Under Reflux Conditions ^b^
1	**3a**	phenyl	3.5	83	8 h, 85%
2	**3b**	4-methoxyphenyl	3	89	6 h, 89%
3	**3c**	4-tolyl	3	82	
4	**3d**	4-(trifluoromethyl)phenyl	3	74	
5	**3e**	4-nitrophenyl	3.5	24	8 h, 24%
6	**3f**	4-chlorophenyl	3	41	6 h, 42%
7	**3g**	4-bromophenyl	3	43	
8	**3h**	3-pyridinyl	3	77	
9	**3i**	2-thiophenyl	2	94	5 h, 85%
10	**3j**	2-furanyl	2	73	
11	**3k**	methyl	7	67	12 h, 66%
12	**3l**	heptyl	6	46	

^a^ Reaction conditions: 4-methoxy enaminonitrile (0.2 mmol, 1.0 equiv.), acyl hydrazides (0.40 mmol, 2.0 equiv.), dry toluene (1.5 mL), microwave heating at 140 °C for the indicated time. ^b^ The reaction was performed under reflux conditions stirred at 120 °C, along with 100 g of 3Å MS in 2.0 mL dry toluene. Isolated yields.

**Table 3:**
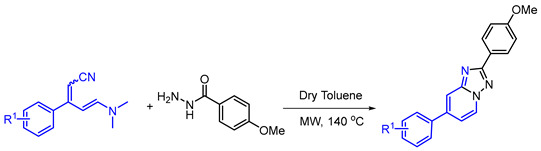
Substrate scope of enaminonitriles ^a^.

Entry No	Compound Code	R^1^	Time (h)	Yield (%)	Under Reflux Conditions ^b^
13	**3m**	H	3	89	
14	**3n**	4-*n*-Pr	4.5	80	7 h, 84%
15	**3o**	4-SMe	3	71	5 h, 54%
16	**3p**	2-NO_2_	7	48	25 h, 47%
17	**3q**	4-F	5	91	
18	**3r**	4-Cl	5	94	
19	**3s**	4-Br	6	90	
20	**3t**	4-I	7	71	12 h, 76%
21	**3u**	2,5-dichloro	7	67	

^a^ Reaction conditions: enaminonitrile (0.20 mmol, 1.0 equiv.), 4-methoxy benzohydrazide (0.40 mmol, 2.0 equiv.), dry toluene (1.5 mL), microwave heating at 140 °C for the indicated time. ^b^ The reaction was performed under reflux conditions at 120 °C, along with 100 g of 3Å MS in 2.0 mL dry toluene. Isolated yields.

## Data Availability

Data are contained within the article and Supplementary Materials.

## Supplementary Materials

The following supporting information can be downloaded at https://www.mdpi.com/article/10.3390/molecules29040894/s1. ^1^H NMR and ^13^C-NMR are available online.
