# Supplementary material for: Microwave-Mediated, Catalyst-Free Synthesis of 1,2,4-Triazolo[1,5-a]pyridines from Enaminonitriles
Source: Molecules. 2024 Feb 18;29(4):894. doi: 10.3390/molecules29040894 (PMC10892893; doi:10.3390/molecules29040894)

Supporting Information

Microwave-Mediated, Catalyst-Free Synthesis of 1,2,4-Triazolo[1,5-*a*]Pyridines from Enaminonitriles

Kwanghee Lee †, Young-Ah Kim †, Chanhyun Jung, Jaeuk Sim, Shanmugam Rajasekar, Jae-Hwan Kwak, Mayavan Viji * and Jae-Kyung Jung *

College of Pharmacy and Medicinal Research Center (MRC), Chungbuk National University, Cheongju 28160, Republic of Korea; kwanghee93@naver.com (K.L.); rladuddk97@naver.com (Y.-A.K.); cksgus9823@naver.com (C.J.); simprog@naver.com (J.S.); rajasekarsrkv@gmail.com (S.R.); jhkwak@chungbuk.ac.kr (J.-H.K.)

* Correspondence: cheviji@gmail.com (M.V.); orgjkjung@chungbuk.ac.kr (J.-K.J.); Tel.: +82-43-261-2635 (J.J); Fax: +82-43-268-2732 (J.J.)

† These authors contributed equally to this work.

Title	Page no
Microwave experiment parameters	2
NMR spectra of synthesized compounds	6

Microwave experiment parameters

At 140 °C, (pressure 0 bar, power 145-160 W)

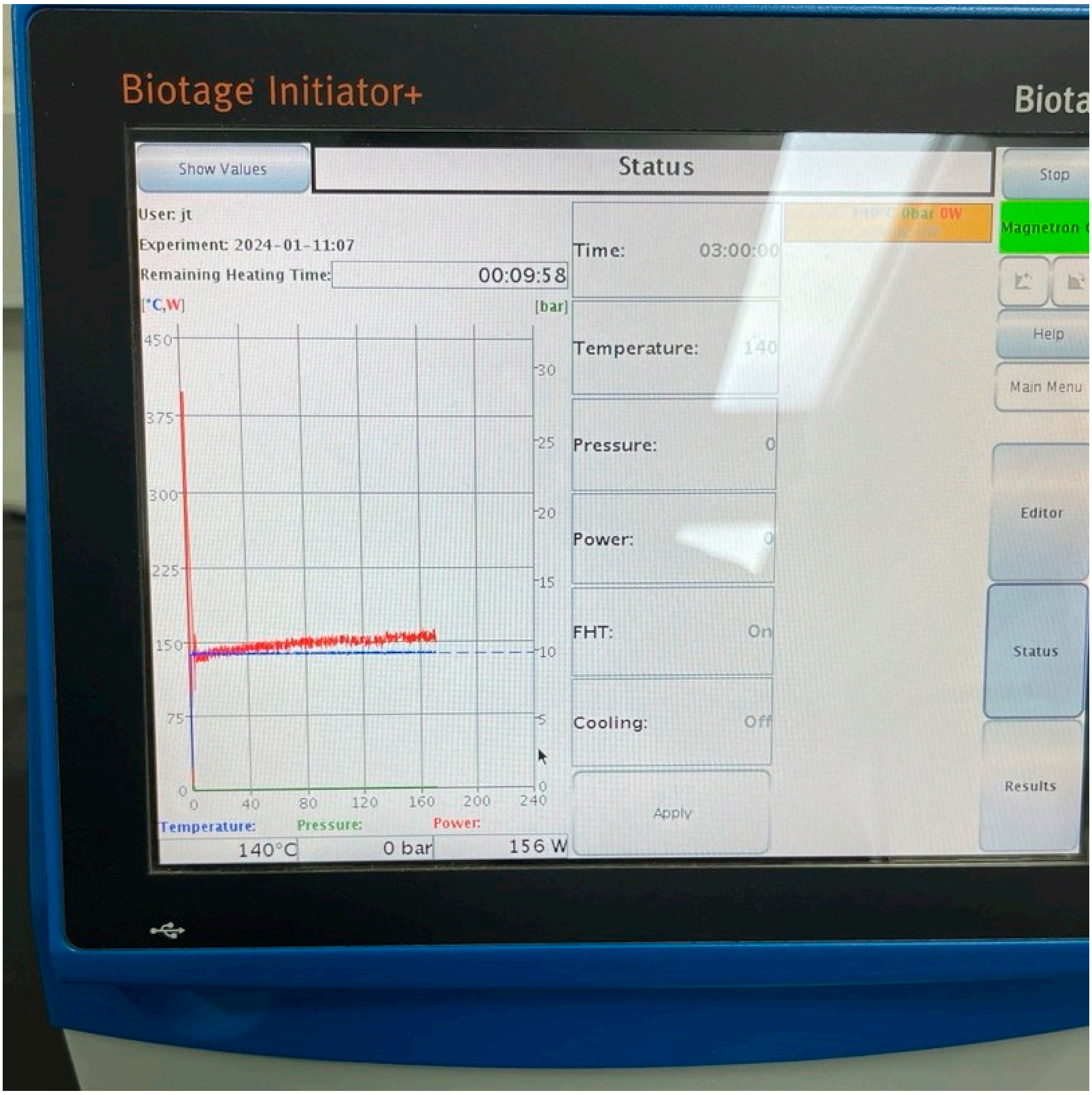

At 160 °C, (pressure 1 bar, power 180-200 W)

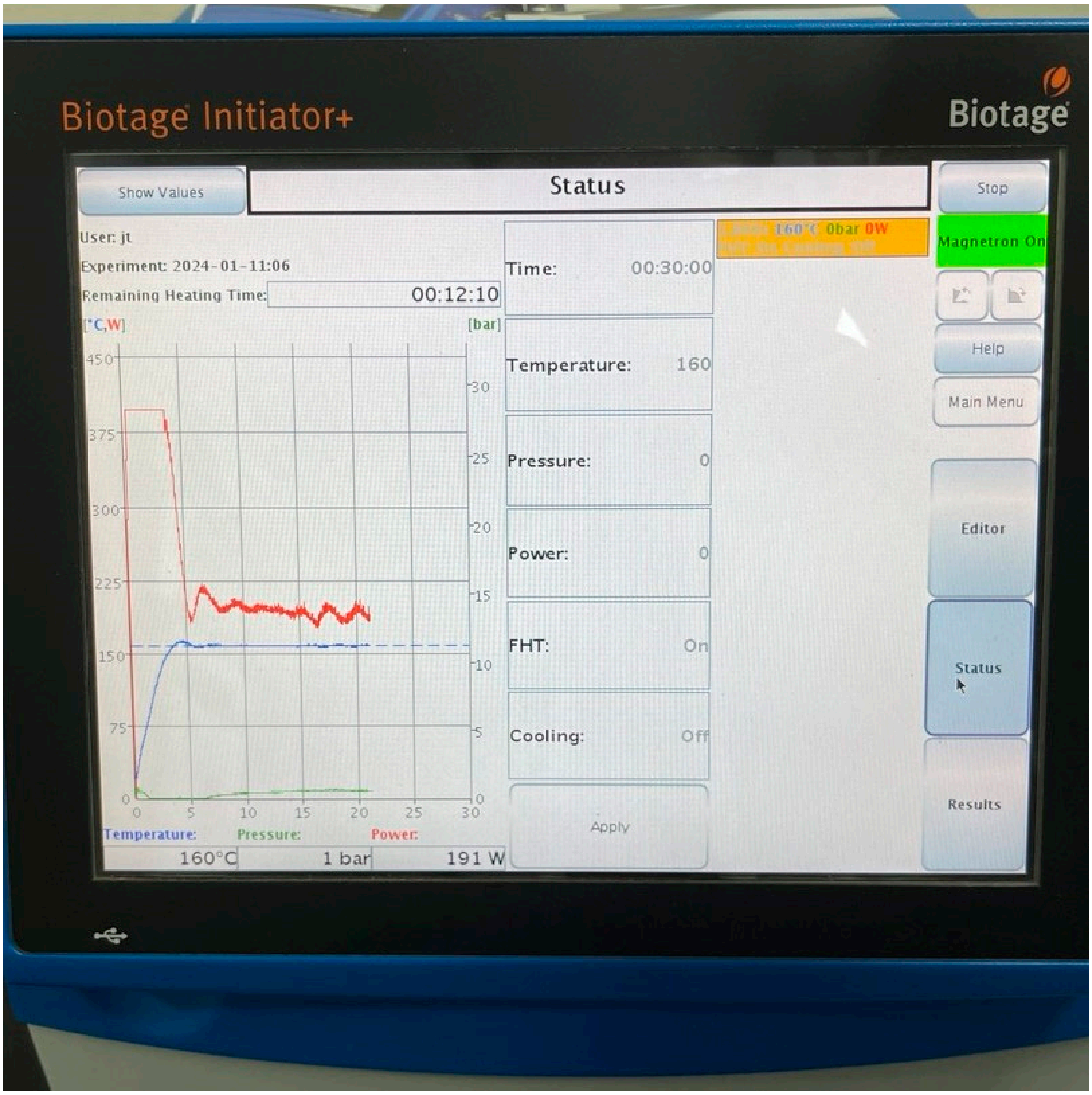

At 180 °C, (pressure 2 bar, power 250-265 W)

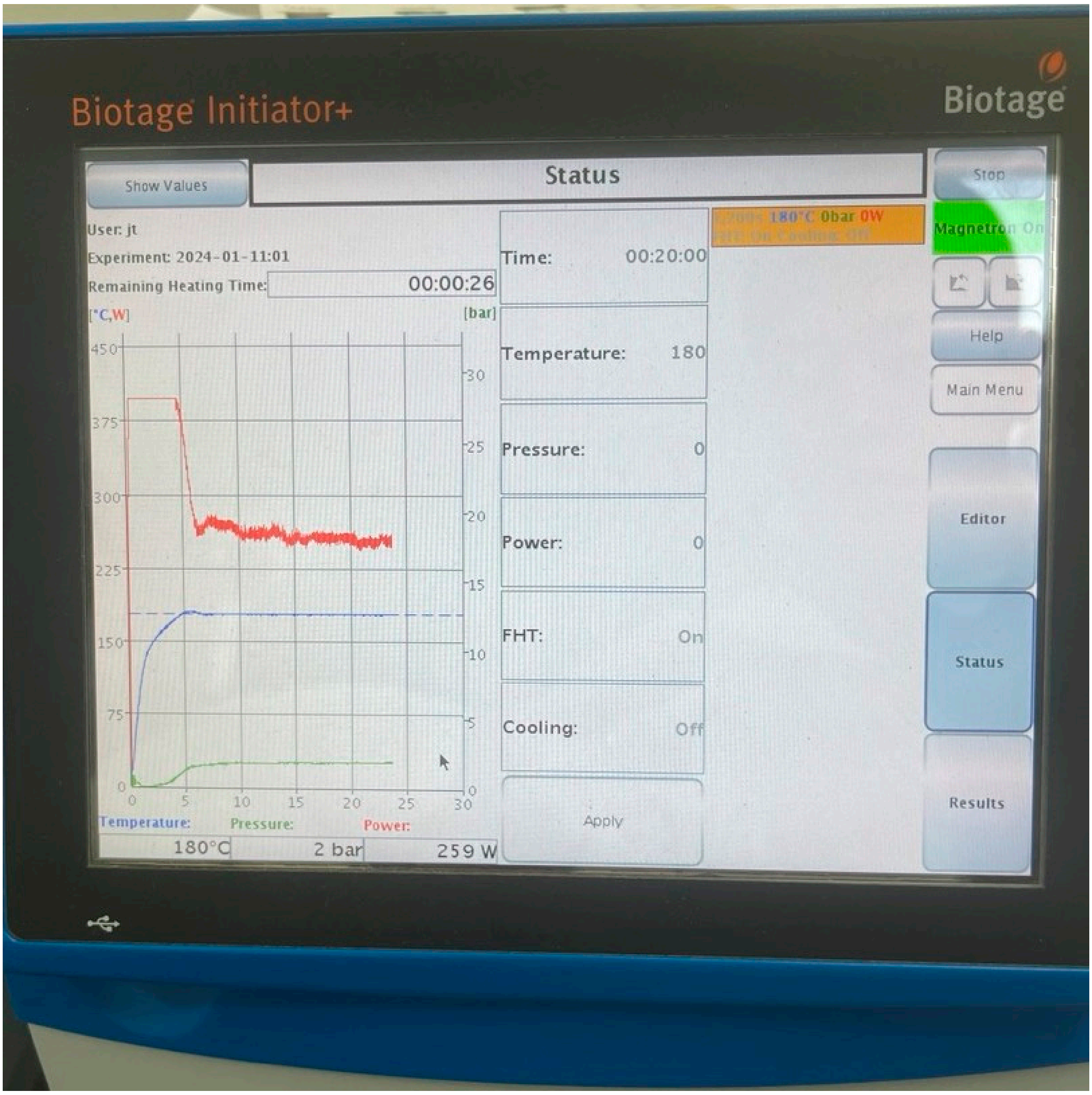

At 140 °C in TBME, (pressure 6 bar, power 175-190 W)

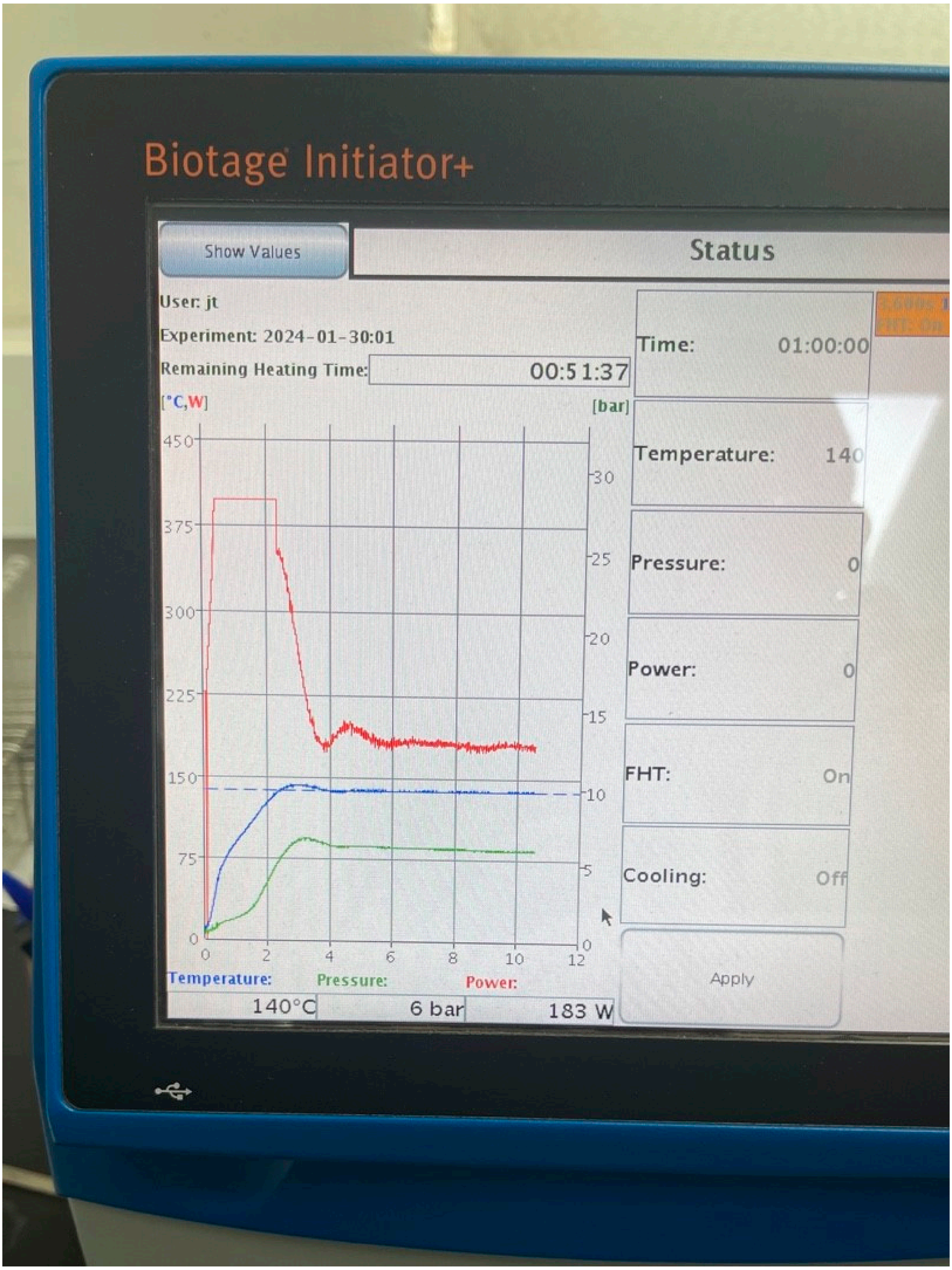

NMR spectra of synthesized compounds

^{1}H (400 MHz, CDCl3) and ^{13}C NMR (100 MHz, CDCl3) of 7-(4-methoxyphenyl)-2-phenyl-[1,2,4]triazolo[1,5-*a*]pyridine (3a)

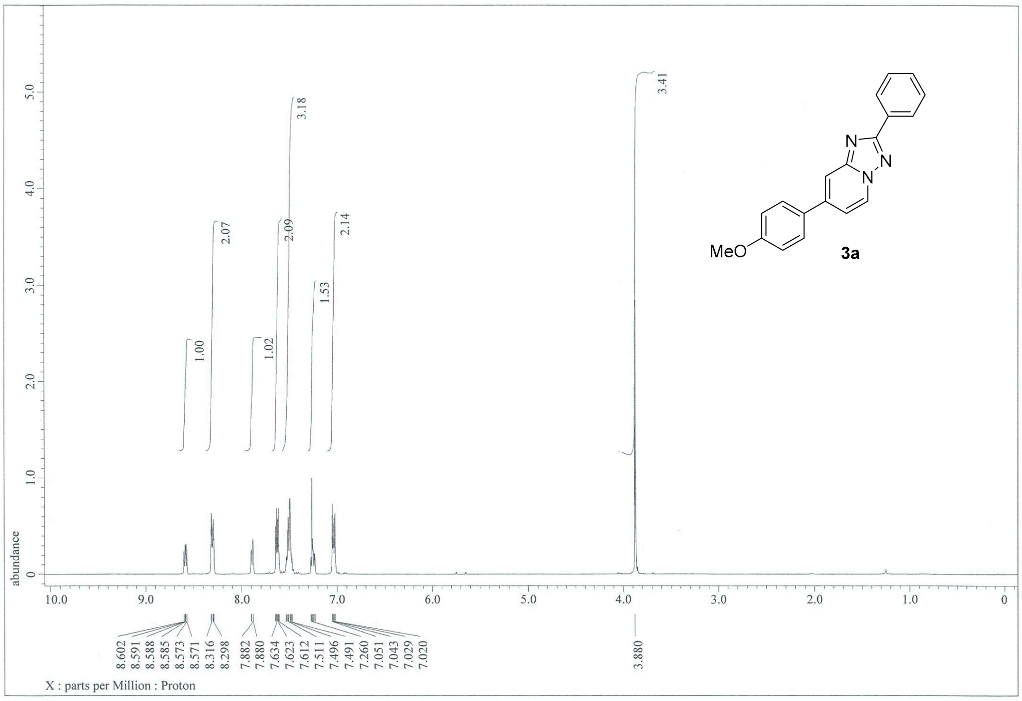

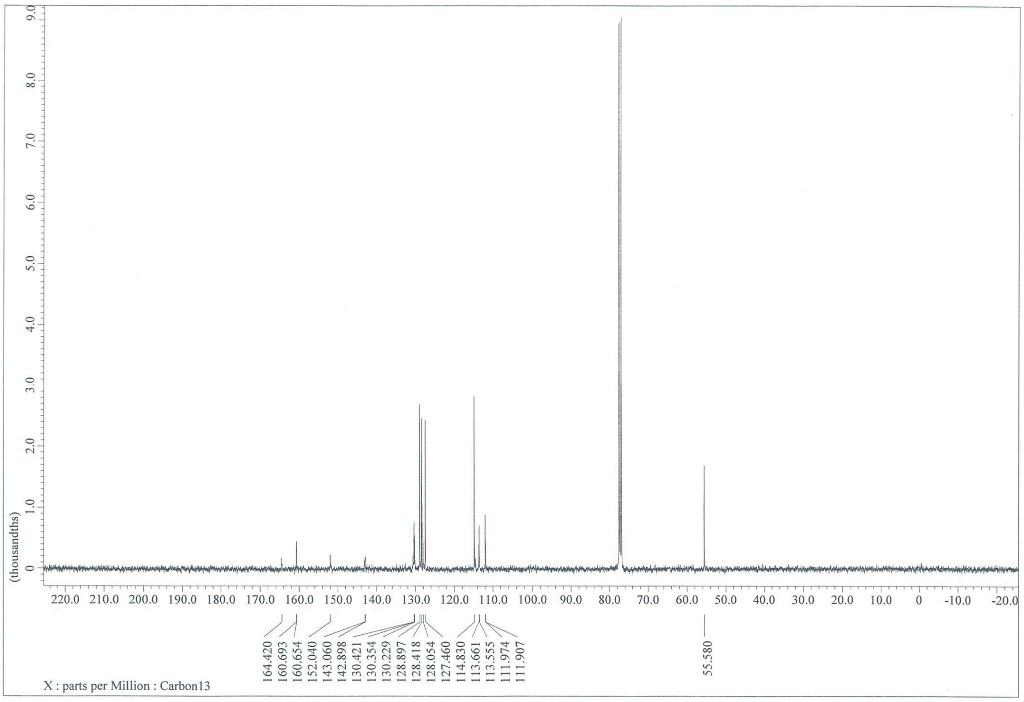

^{1}H (400 MHz, $CDCl_3$) and ^{13}C NMR (100 MHz, $CDCl_3$) of 2,7-bis(4-methoxyphenyl)-[1,2,4]triazolo[1,5-a]pyridine (3b)

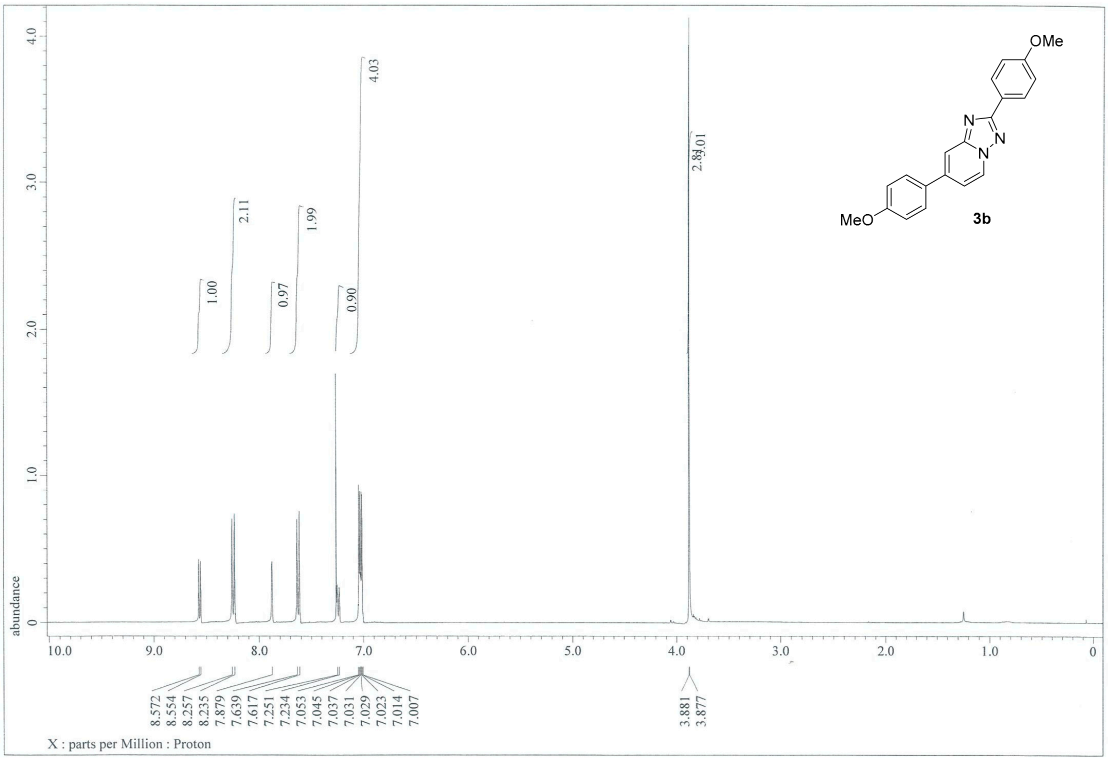

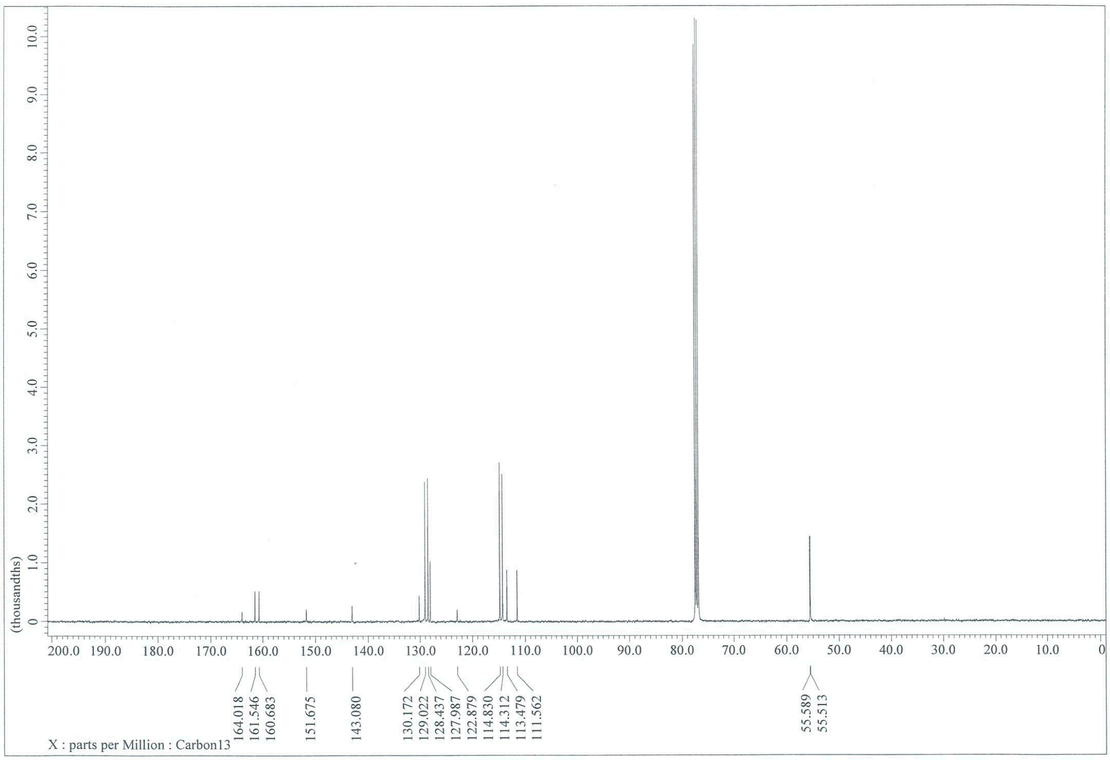

^{1}H (400 MHz, $CDCl_3$) and ^{13}C NMR (100 MHz, $CDCl_3$) of 7-(4-methoxyphenyl)-2-(p-tolyl)-[1,2,4]triazolo[1,5-*a*]pyridine (3c)

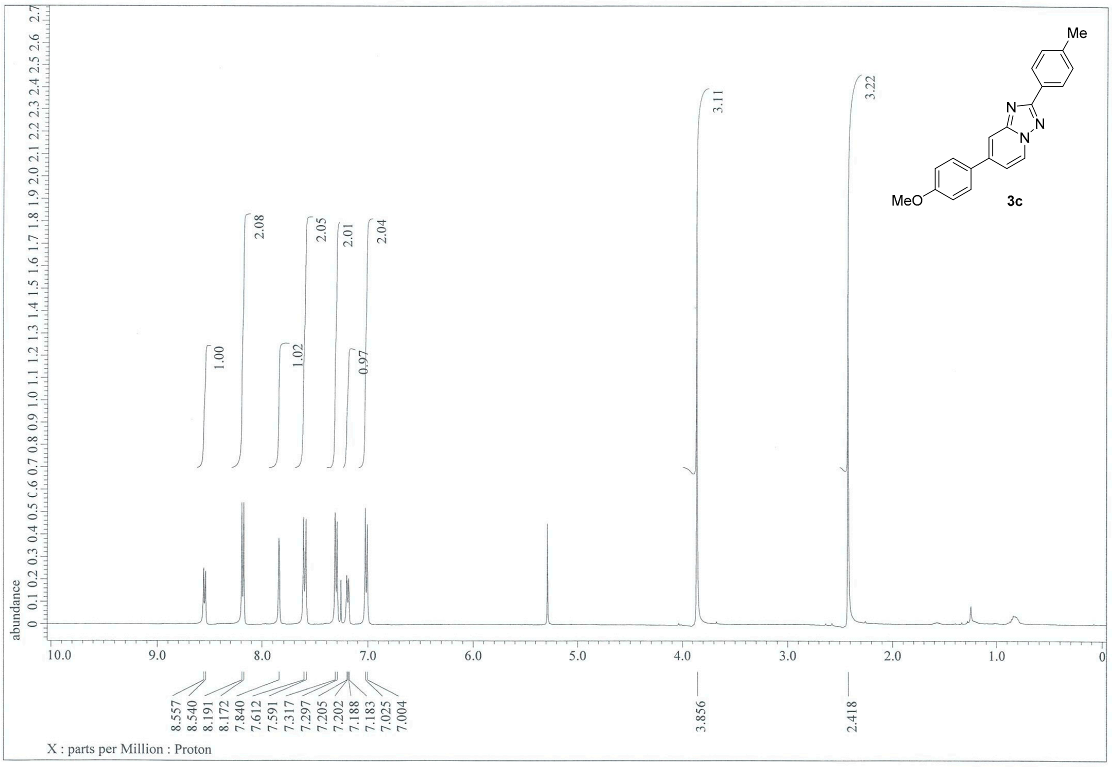

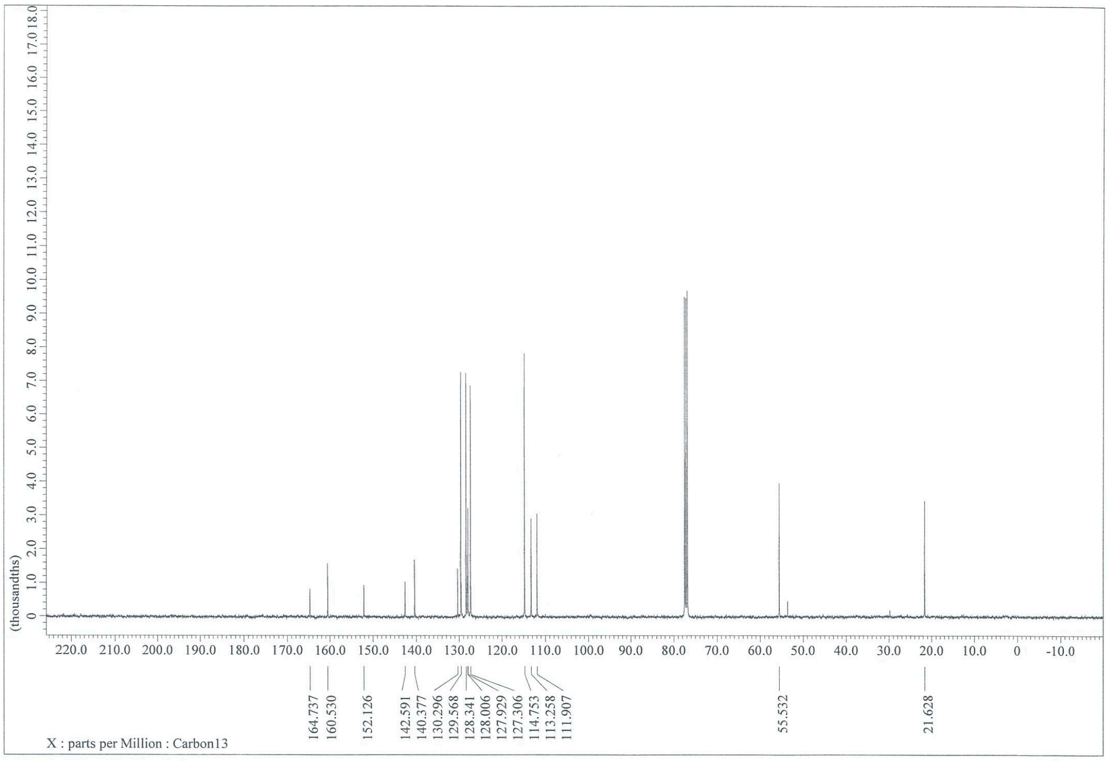

^{1}H (400 MHz, CDCl3) and ^{13}C NMR (100 MHz, CDCl3) of 7-(4-methoxyphenyl)-2-(4-(trifluoromethyl)phenyl)-[1,2,4]triazolo[1,5-*a*]pyridine (3d)

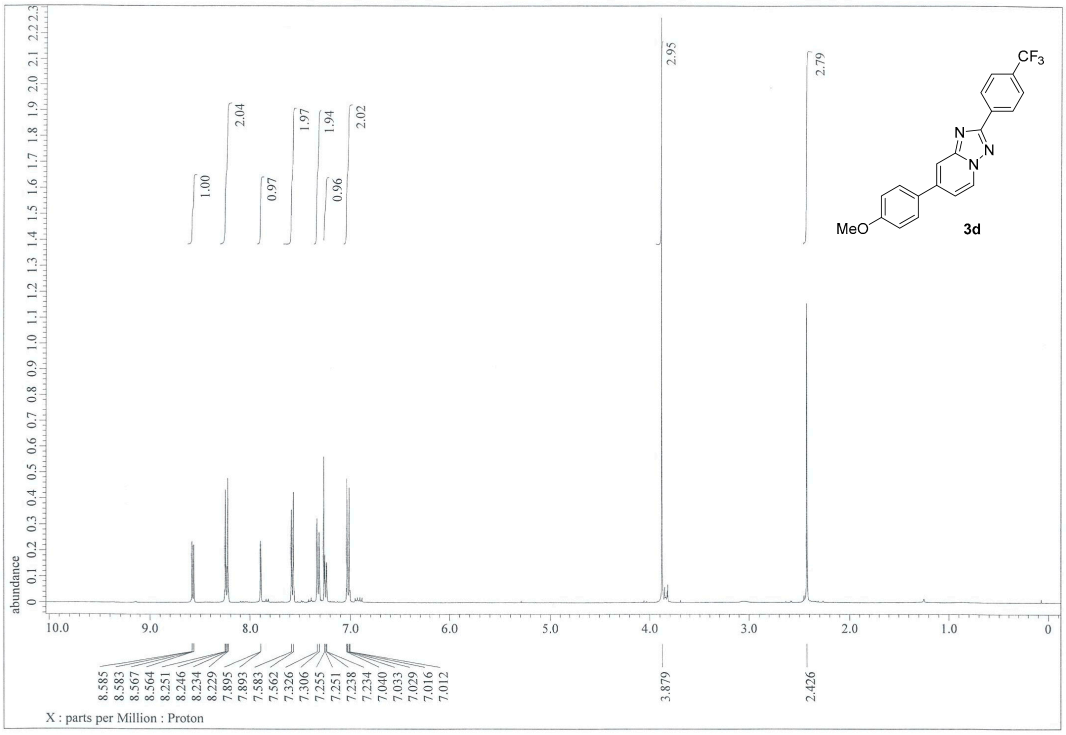

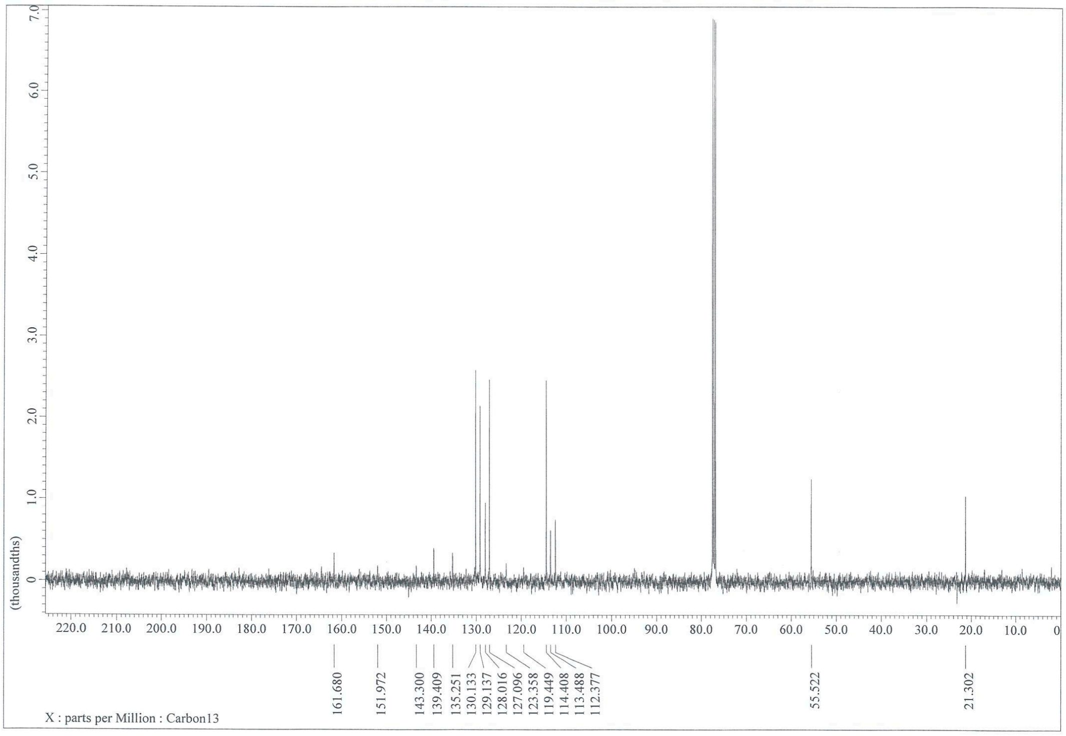

^{19}F NMR (376 MHz, $CDCl_3$) of 7-(4-methoxyphenyl)-2-(4-(trifluoromethyl)phenyl)-[1,2,4]triazolo[1,5-*a*]pyridine (3d)

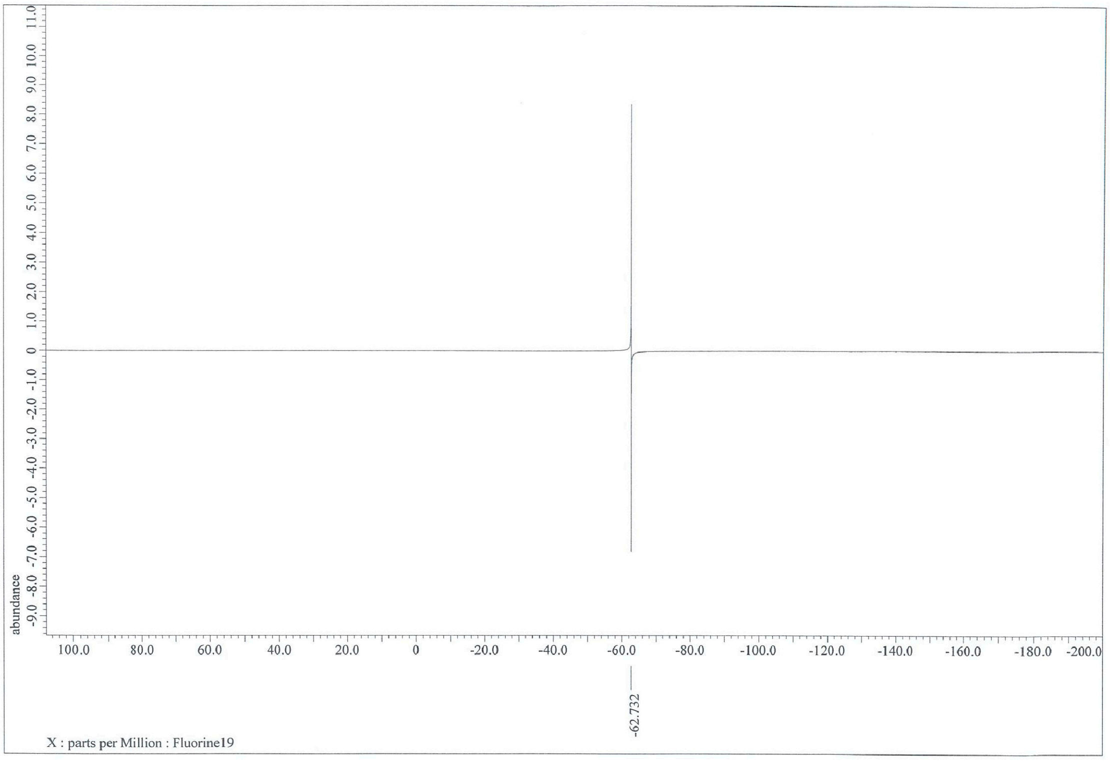

^{1}H (400 MHz, CDCl3) and ^{13}C NMR (100 MHz, DMSO-*d6*) of 7-(4-methoxyphenyl)-2-(4-nitrophenyl)-[1,2,4]triazolo[1,5-*a*]pyridine (3e)

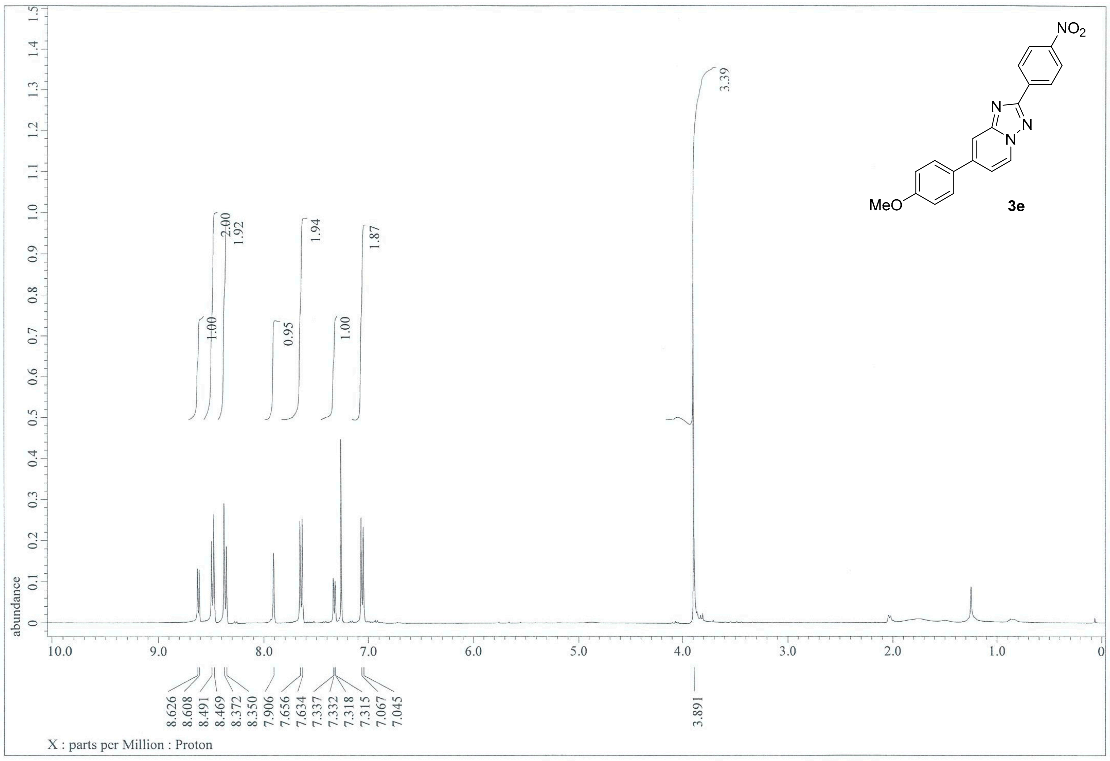

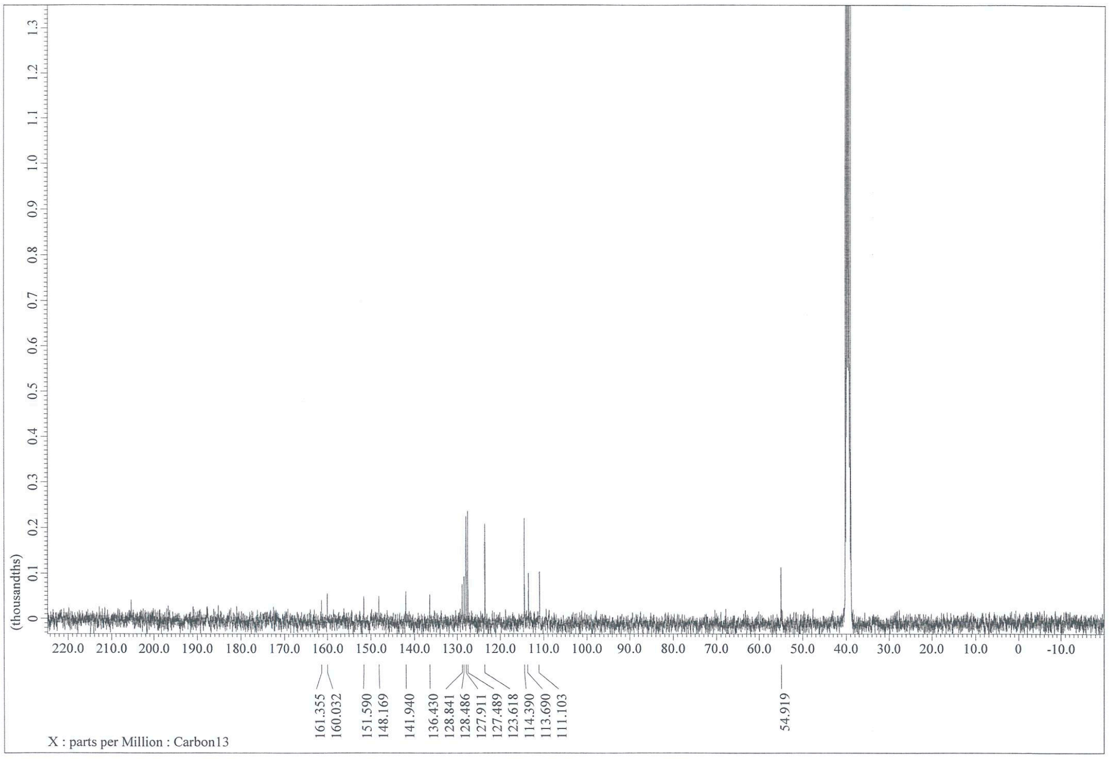

^{1}H (400 MHz, $CDCl_3$) and ^{13}C NMR (100 MHz, $CDCl_3$) of 2-(4-chlorophenyl)-7-(4-methoxyphenyl)-[1,2,4]triazolo[1,5-*a*]pyridine (3f)

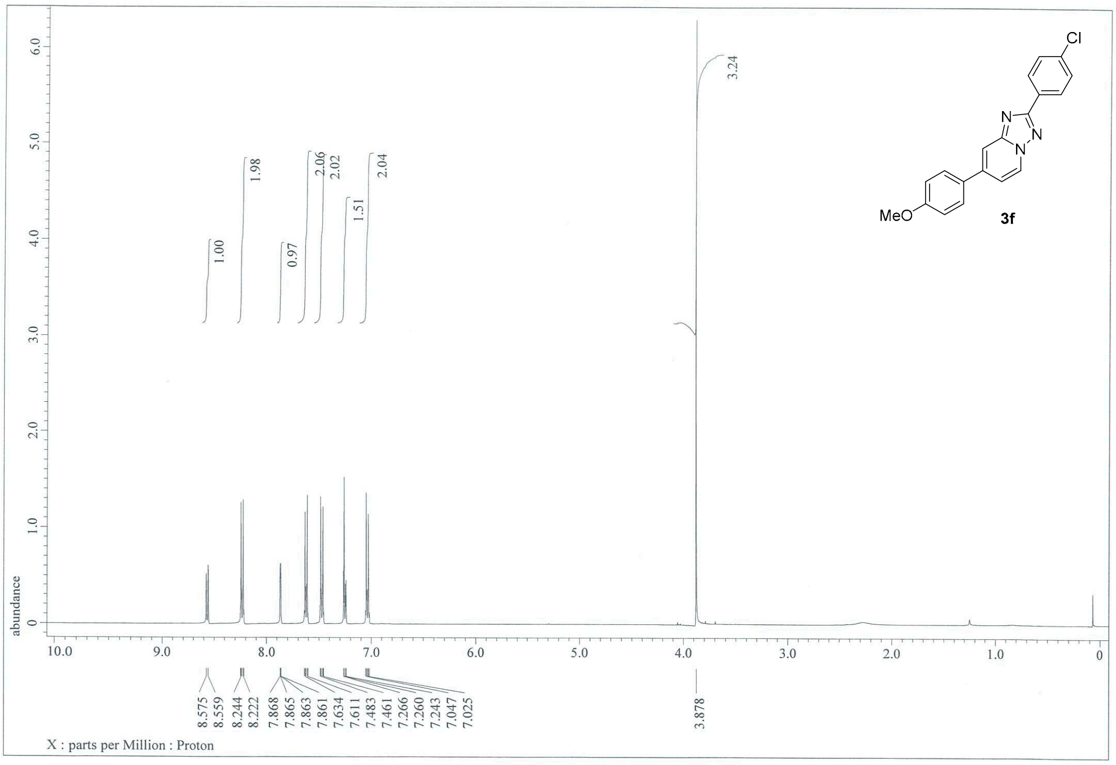

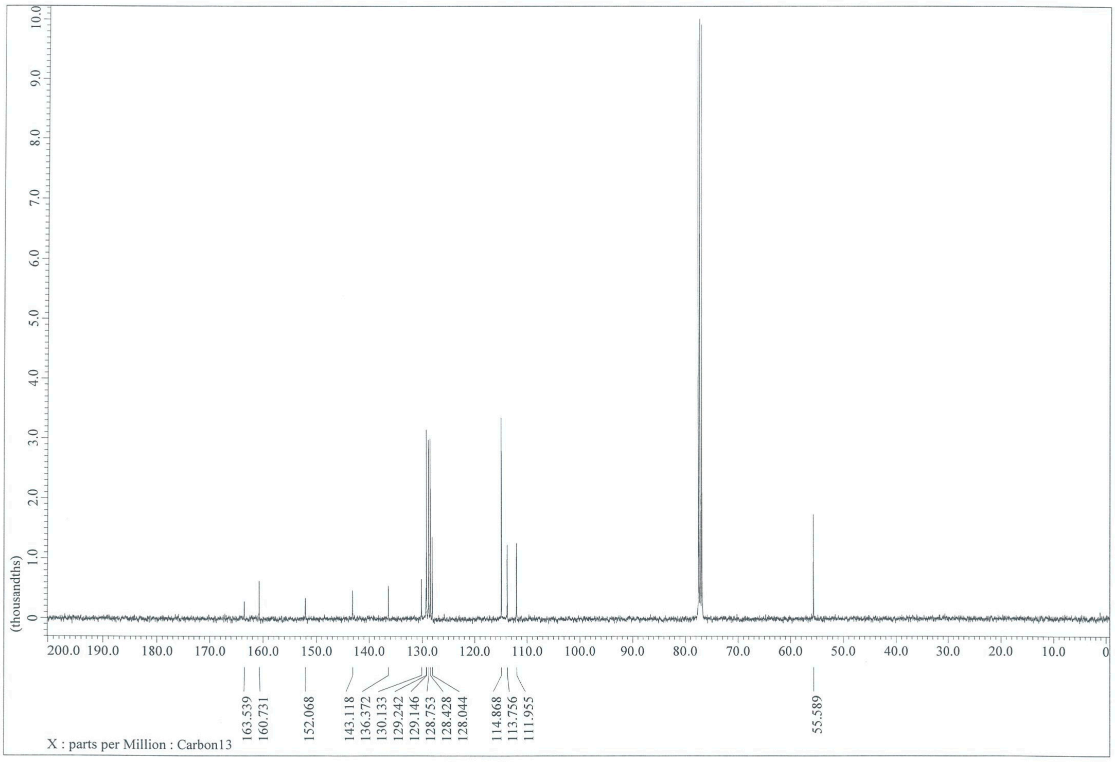

^1H (400 MHz, $CDCl_3$) and ^{13}C NMR (100 MHz, DMSO-*d$_6$*) of 2-(4-bromophenyl)-7-(4-methoxyphenyl)-[1,2,4]triazolo[1,5-*a*]pyridine (3g)

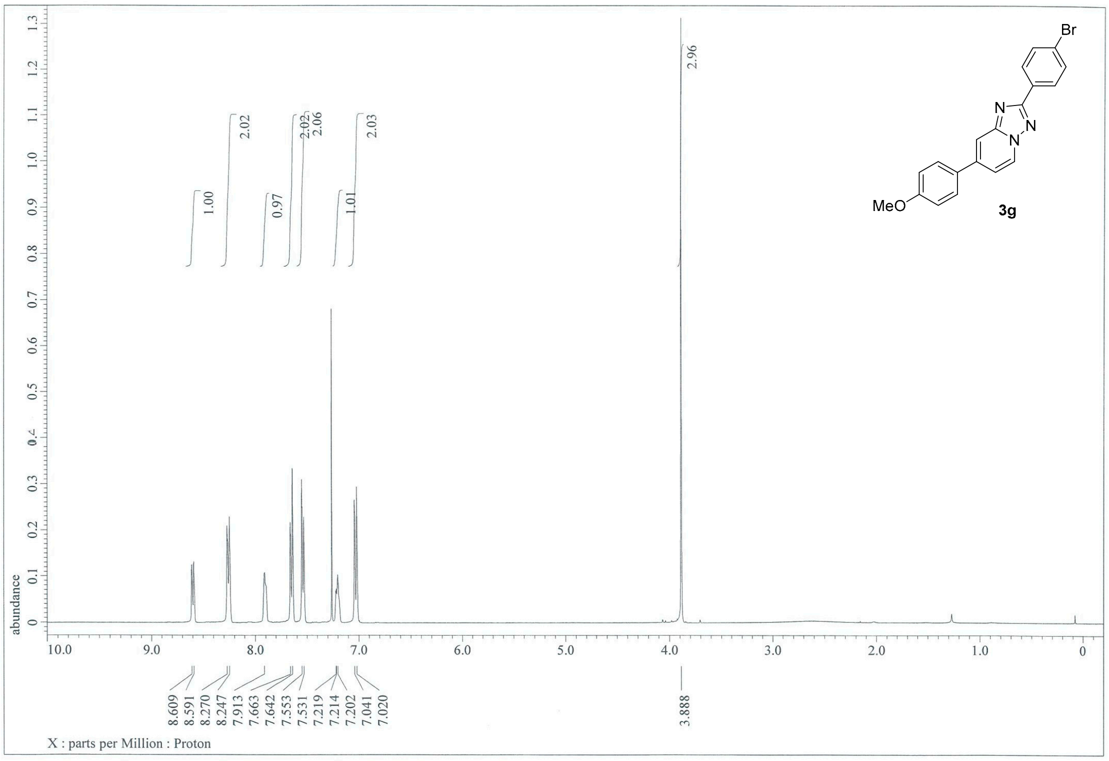

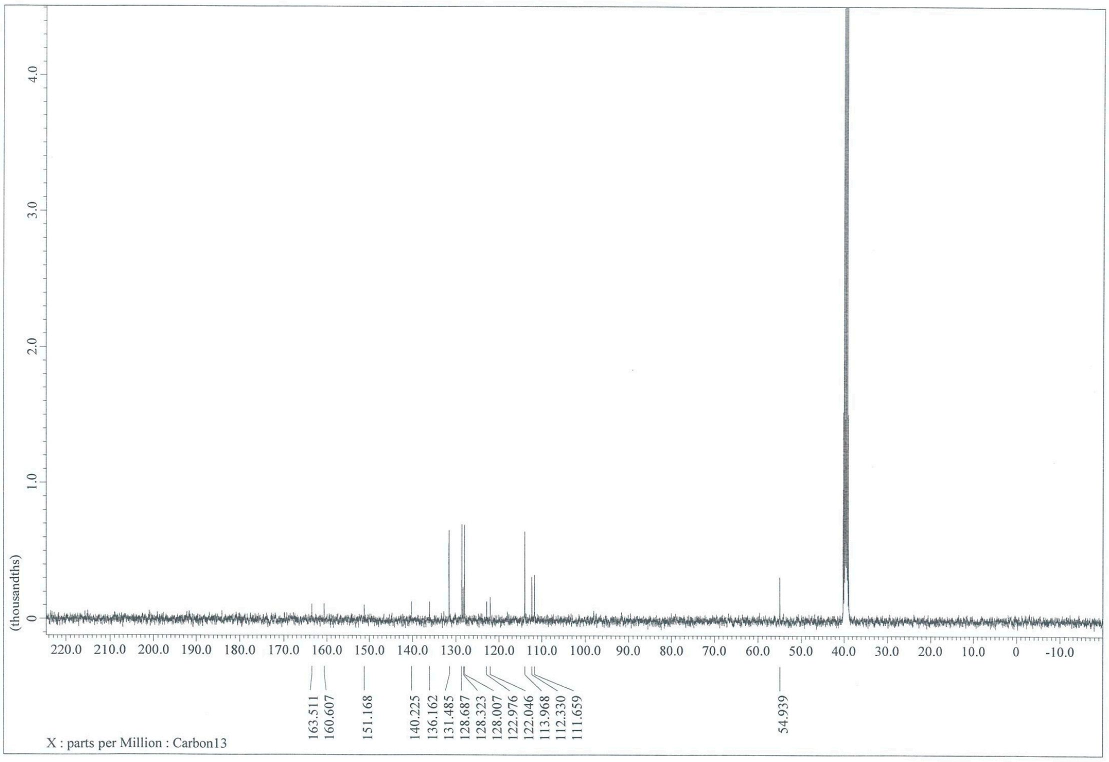

^{1}H (400 MHz, CDCl3) and ^{13}C NMR (100 MHz, CDCl3) of 7-(4-methoxyphenyl)-2-(pyridin-3-yl)-[1,2,4]triazolo[1,5-*a*]pyridine (3h)

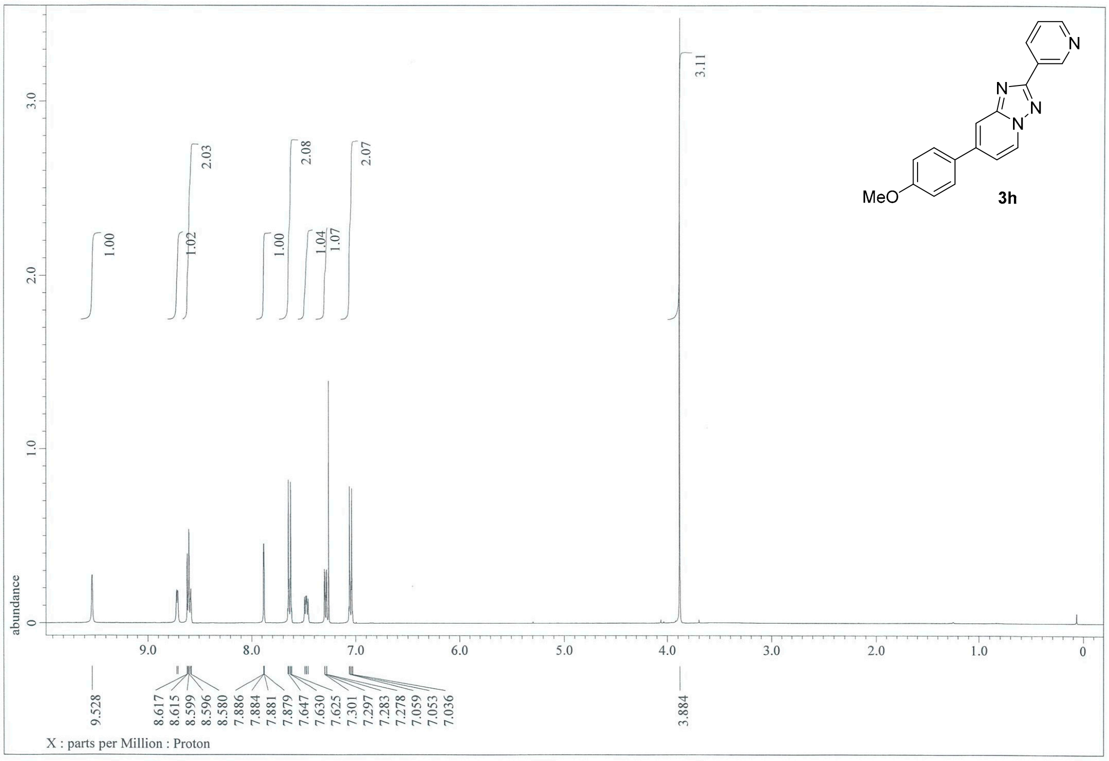

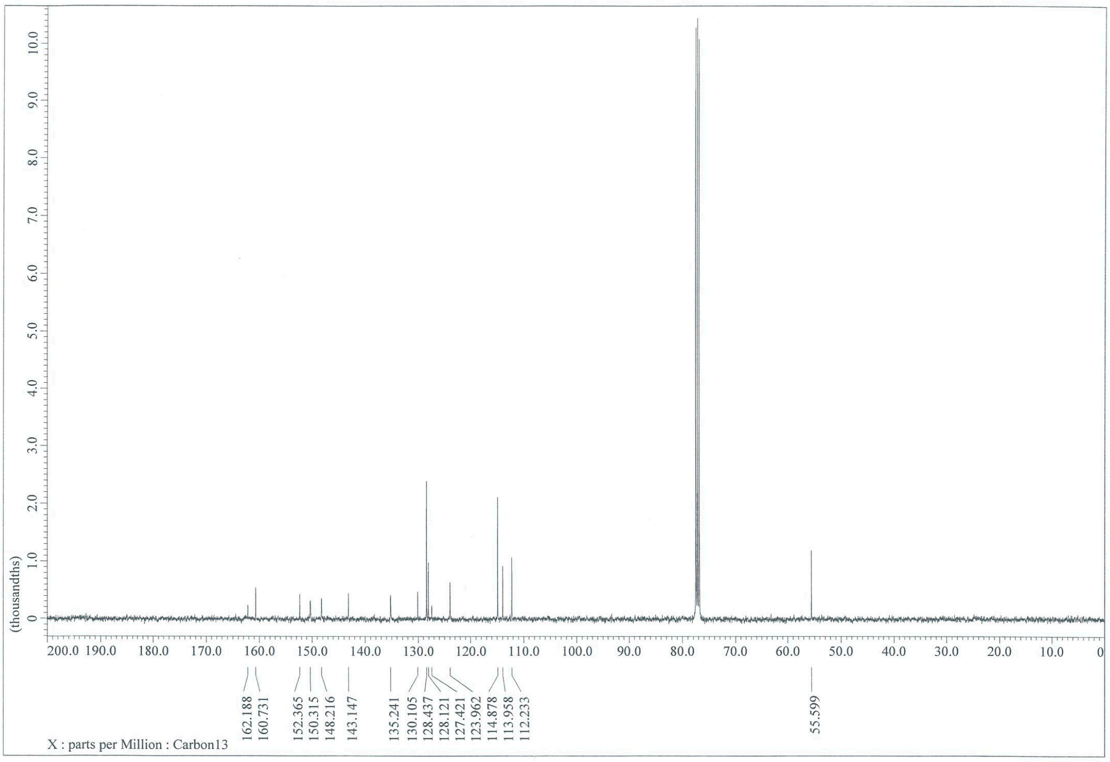

^1H (400 MHz, $CDCl_3$) and ^{13}C NMR (100 MHz, $CDCl_3$) of 7-(4-methoxyphenyl)-2-(thiophen-2-yl)-[1,2,4]triazolo[1,5-*a*]pyridine (3i)

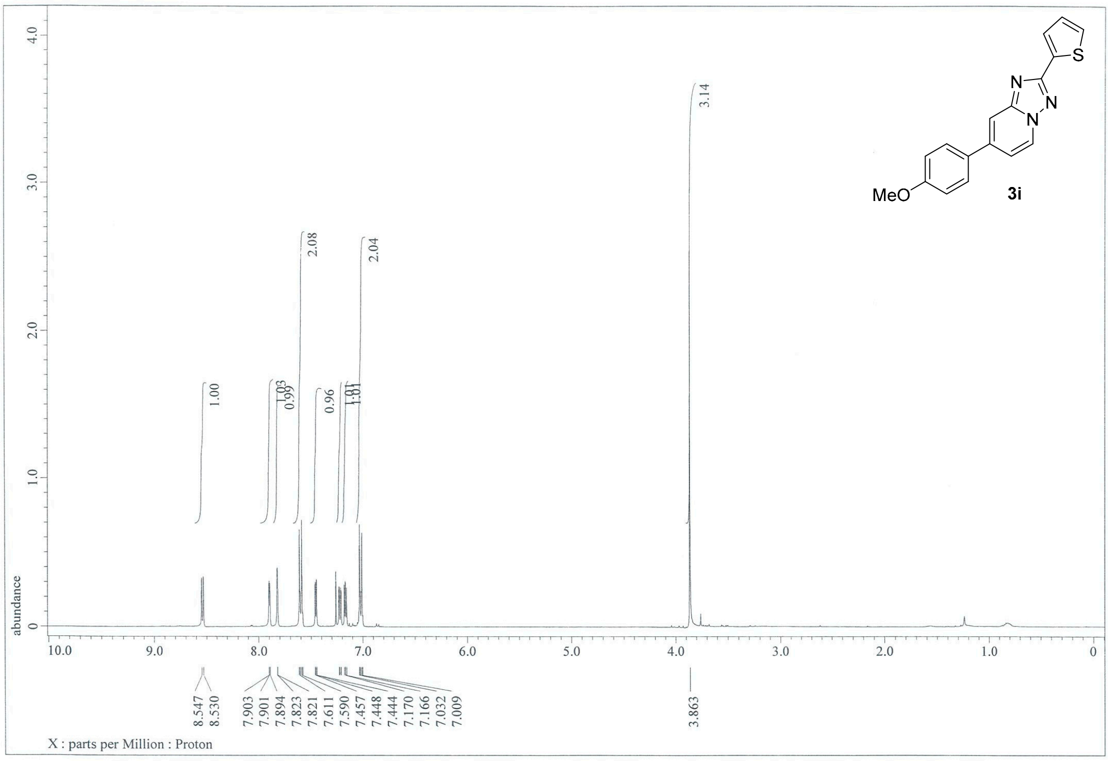

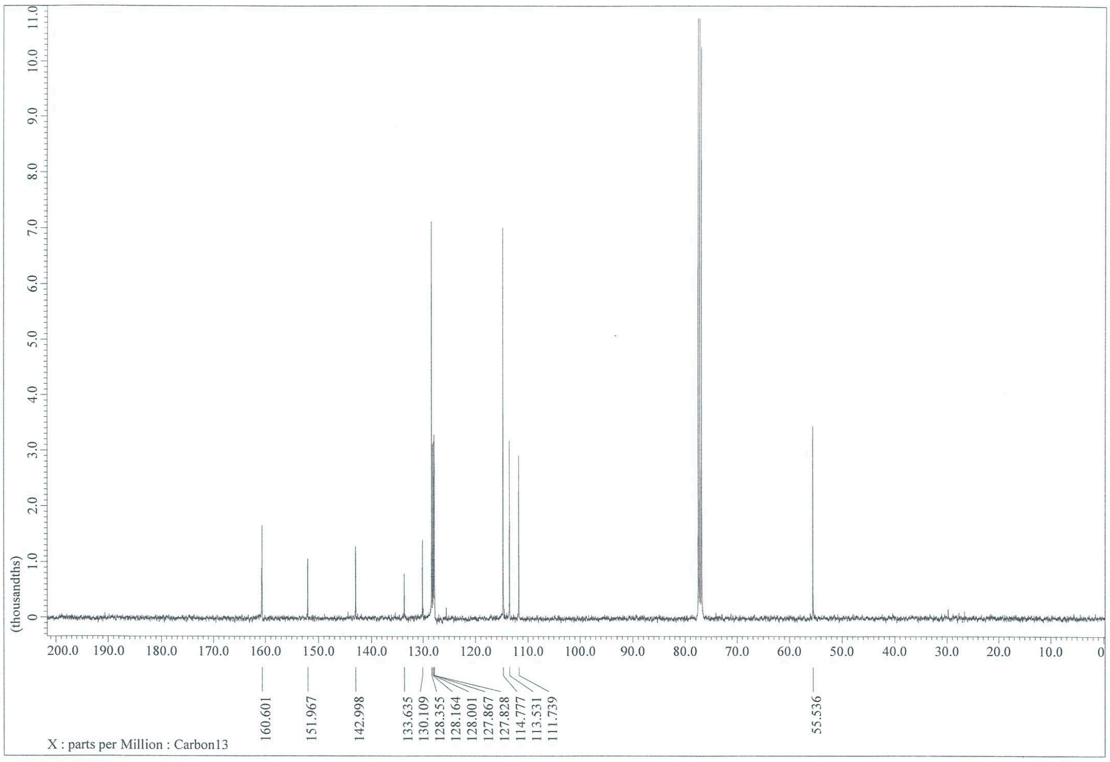

^{1}H (400 MHz, $CDCl_3$) and ^{13}C NMR (100 MHz, $CDCl_3$) of 2-(furan-2-yl)-7-(4-methoxyphenyl)-[1,2,4]triazolo[1,5-*a*]pyridine (3j)

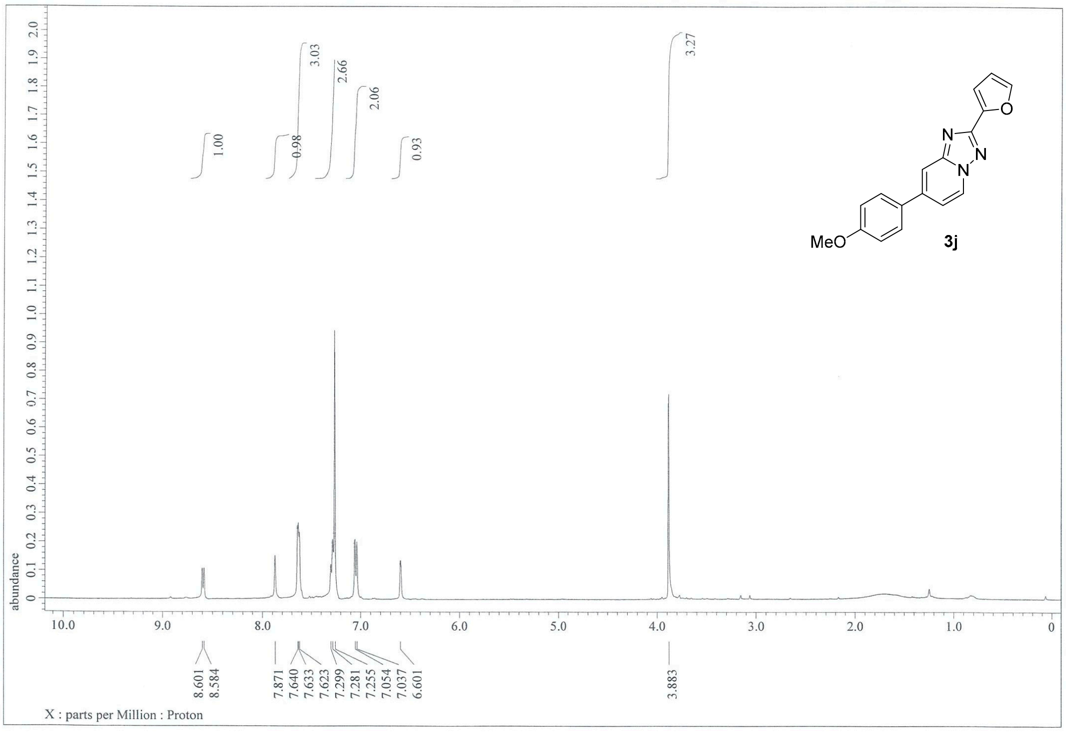

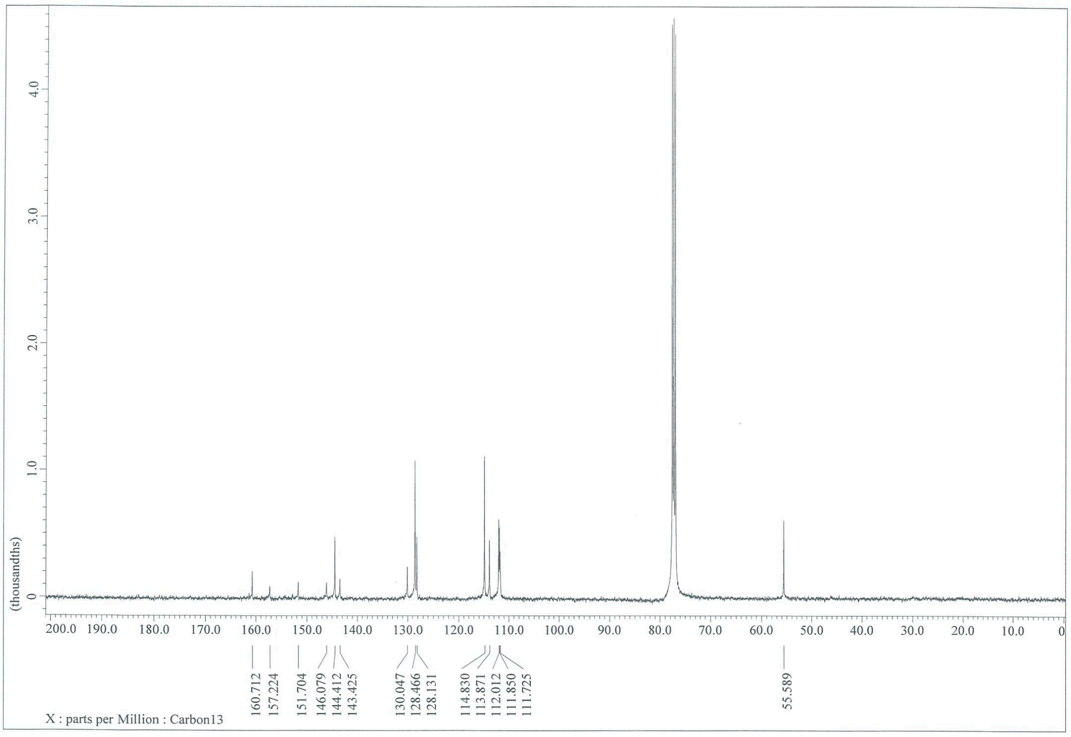

^{1}H (400 MHz, $CDCl_3$) and ^{13}C NMR (100 MHz, $CDCl_3$) of 7-(4-methoxyphenyl)-2-methyl-[1,2,4]triazolo[1,5-*a*]pyridine (3k)

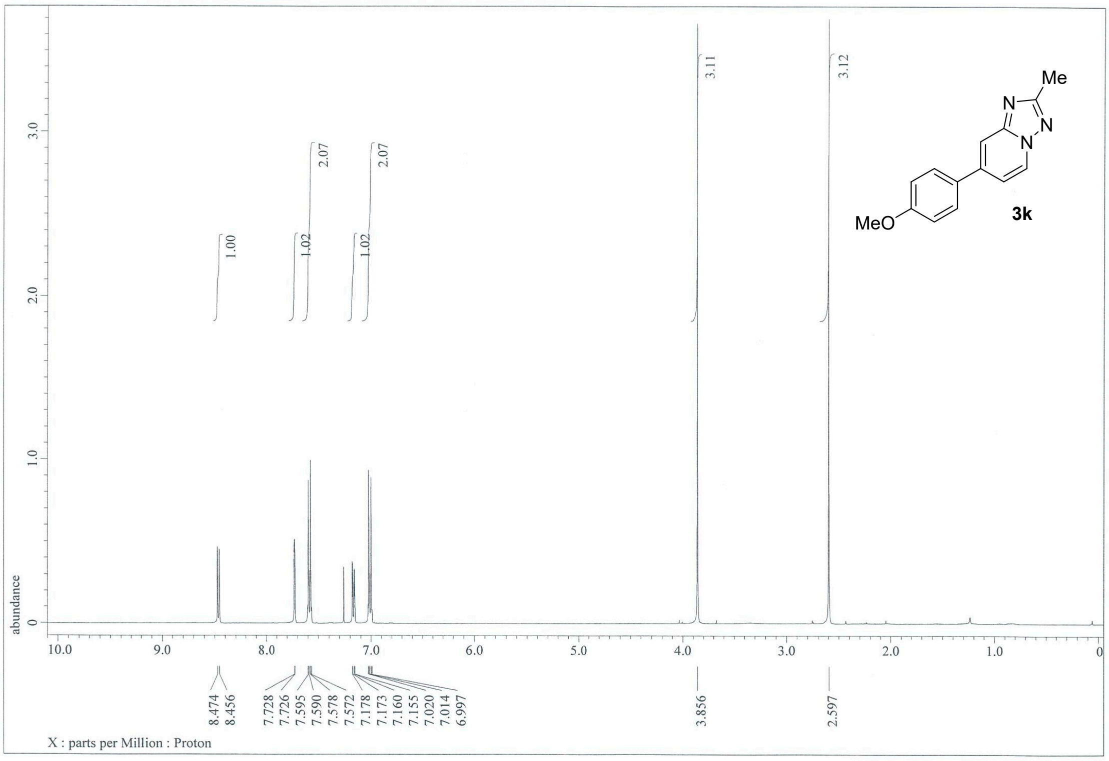

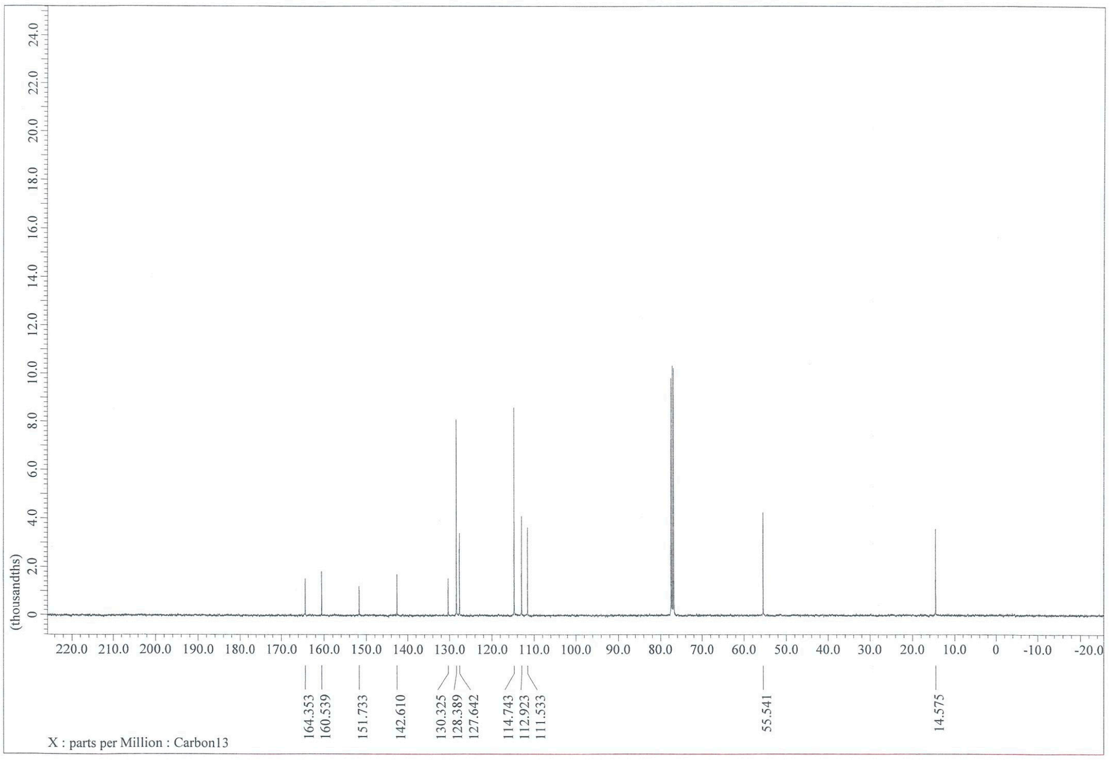

^{1}H (400 MHz, CDCl$_3$) and ^{13}C NMR (100 MHz, CDCl$_3$) of 2-heptyl-7-(4-methoxyphenyl)-[1,2,4]triazolo[1,5-*a*]pyridine (3l)

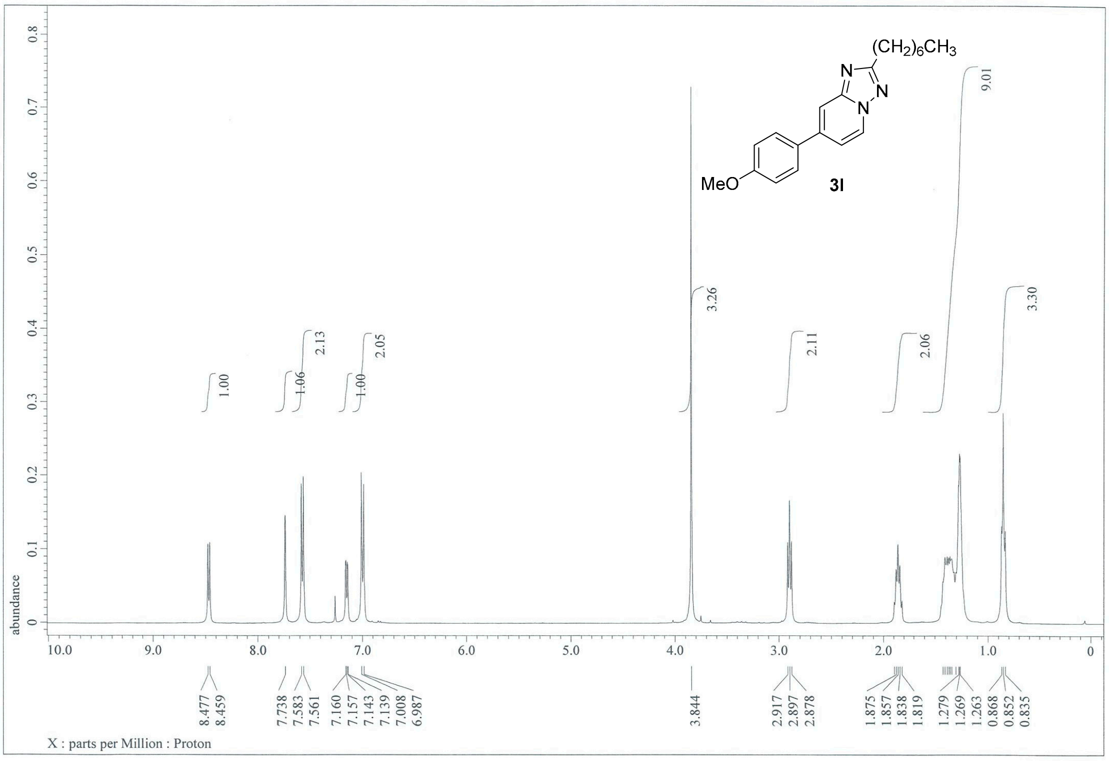

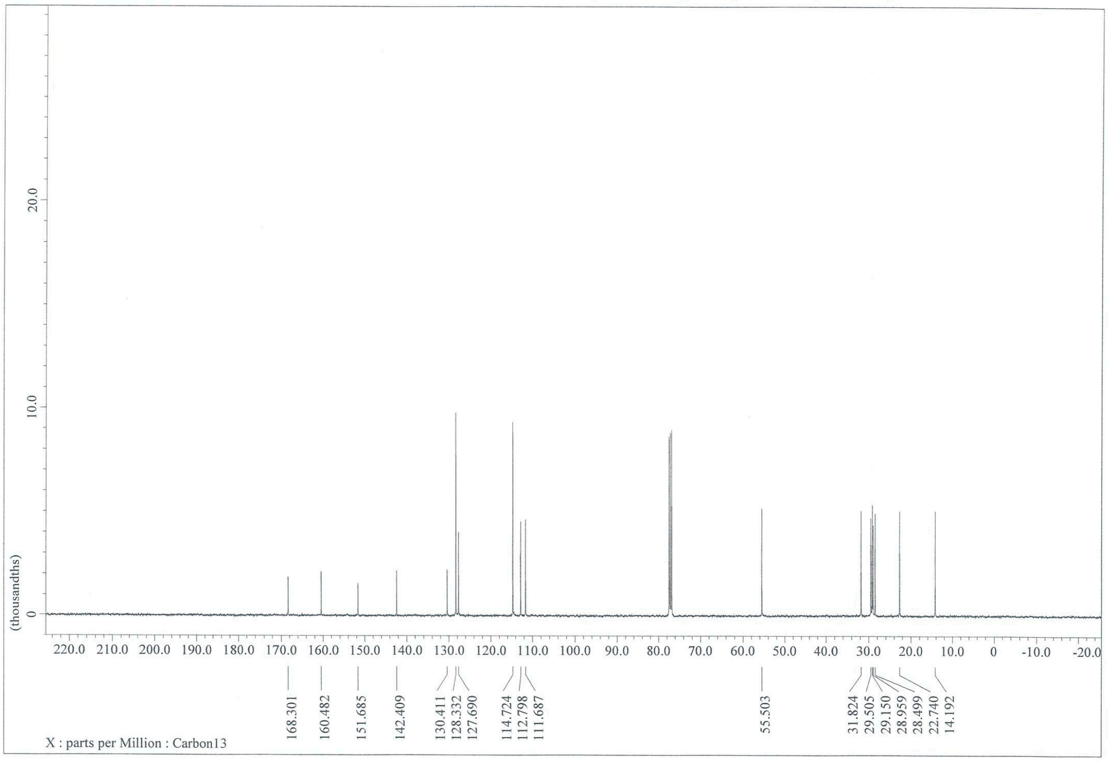

^{1}H (400 MHz, $CDCl_3$) and ^{13}C NMR (100 MHz, $CDCl_3$) of 2-(4-methoxyphenyl)-7-phenyl-[1,2,4]triazolo[1,5-*a*]pyridine (3m)

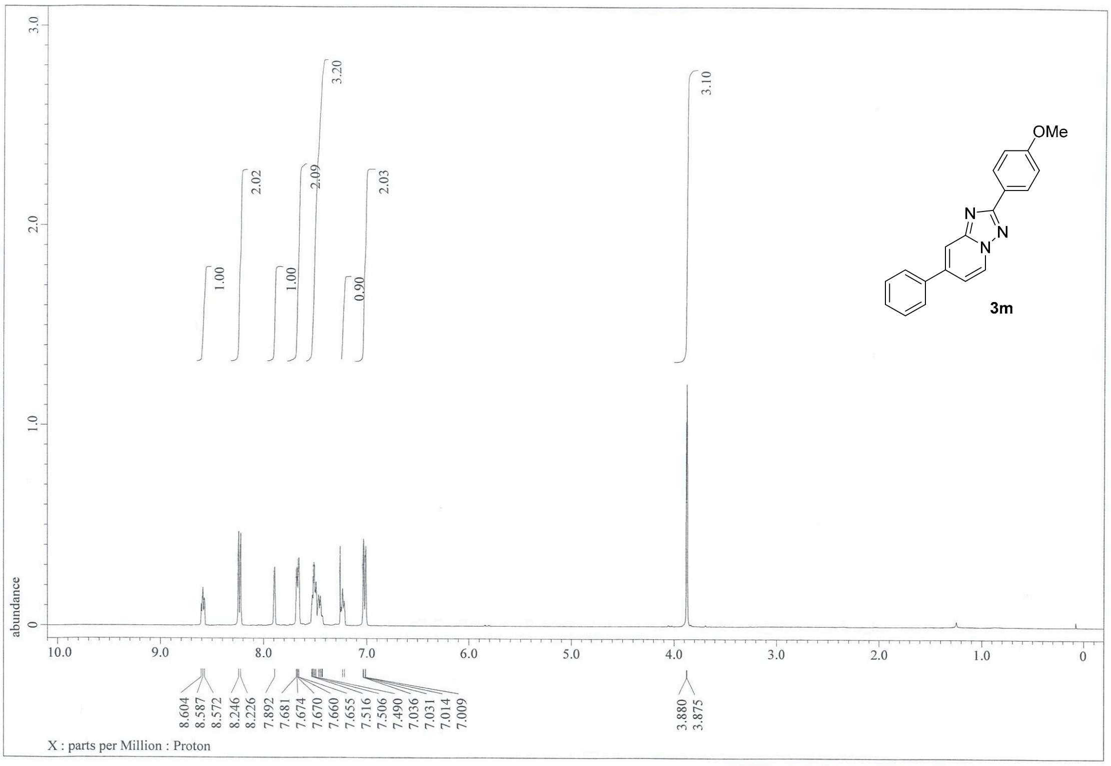

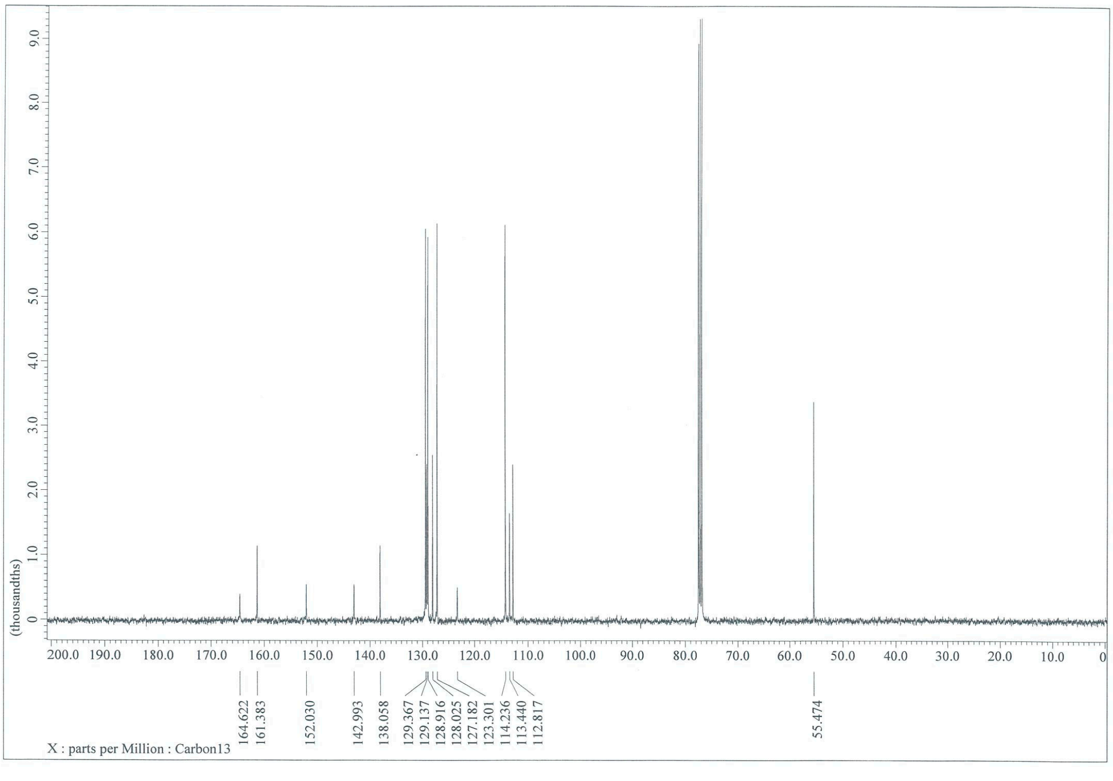

^{1}H (400 MHz, $CDCl_3$) and ^{13}C NMR (100 MHz, $CDCl_3$) of 2-(4-methoxyphenyl)-7-(4-propylphenyl)-[1,2,4]triazolo[1,5-*a*]pyridine (3n)

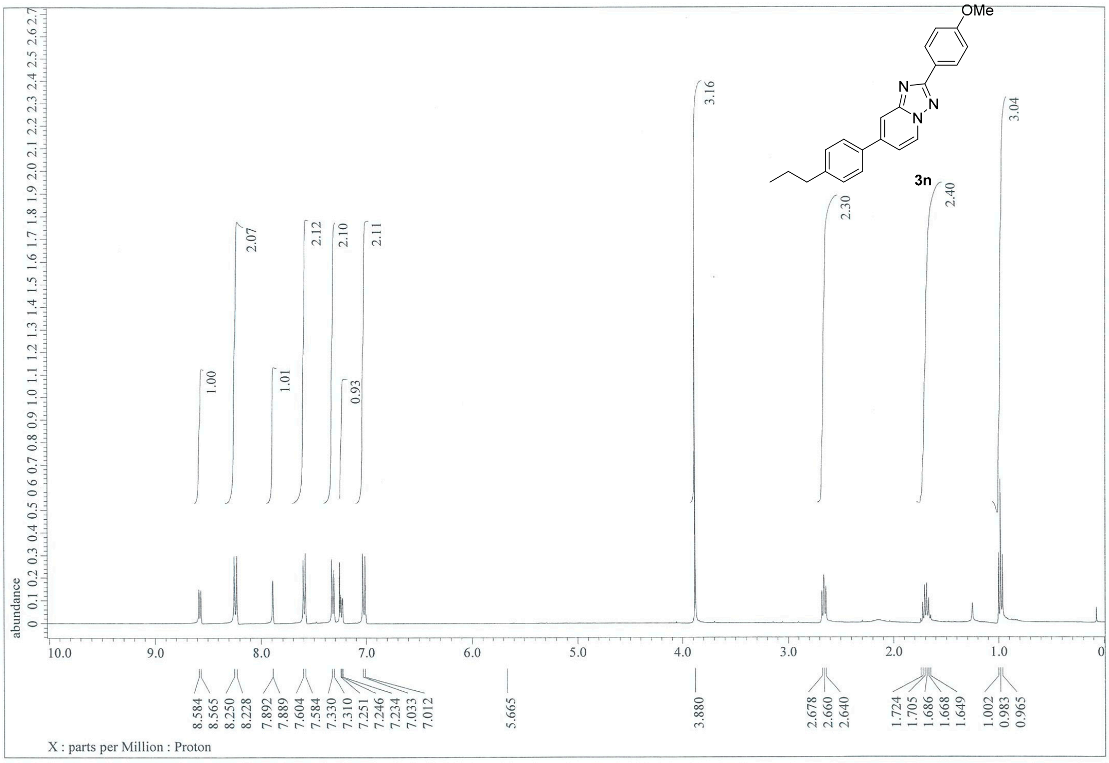

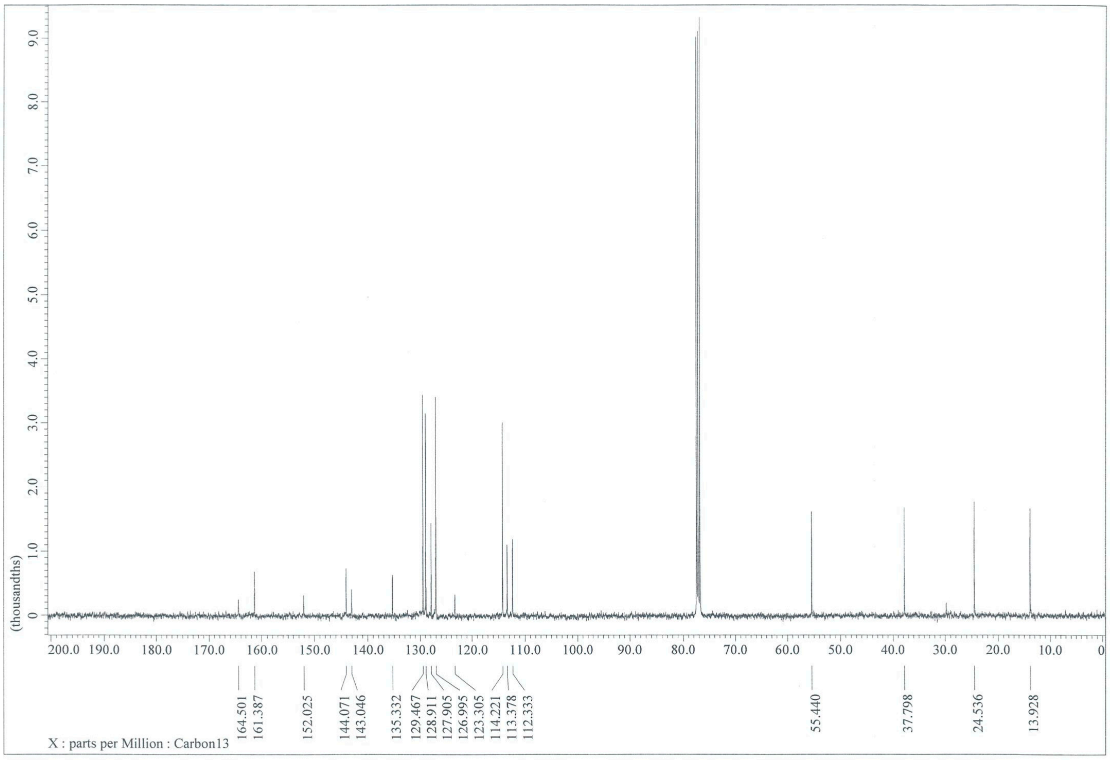

^{1}H (400 MHz, $CDCl_3$) and ^{13}C NMR (100 MHz, $CDCl_3$) of 2-(4-methoxyphenyl)-7-(4-(methylthio)phenyl)-[1,2,4]triazolo[1,5-*a*]pyridine (3o)

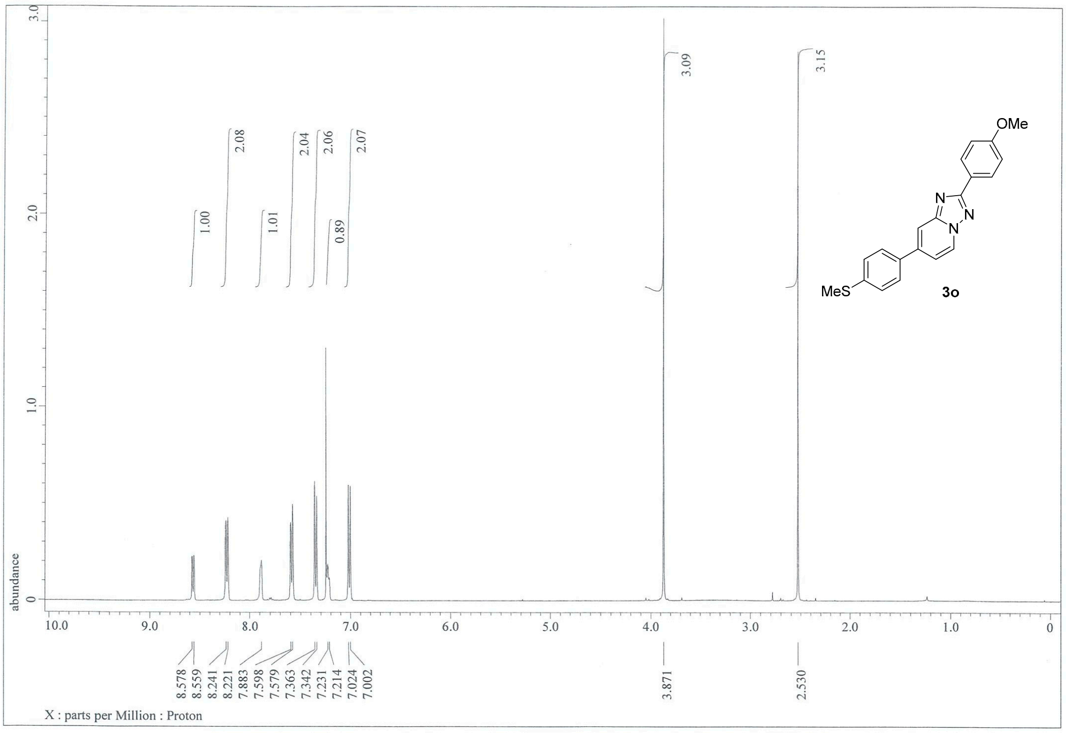

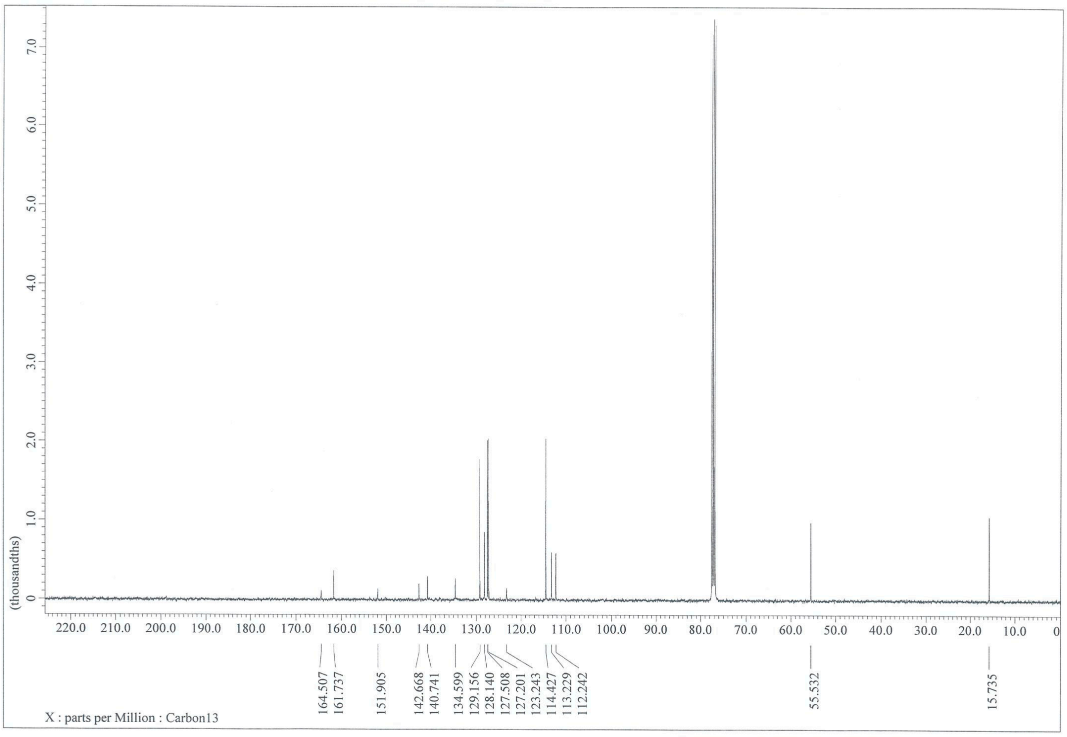

^{1}H (400 MHz, $CDCl_3$) and ^{13}C NMR (100 MHz, $CDCl_3$) of 2-(4-methoxyphenyl)-7-(2-nitrophenyl)-[1,2,4]triazolo[1,5-*a*]pyridine (3p)

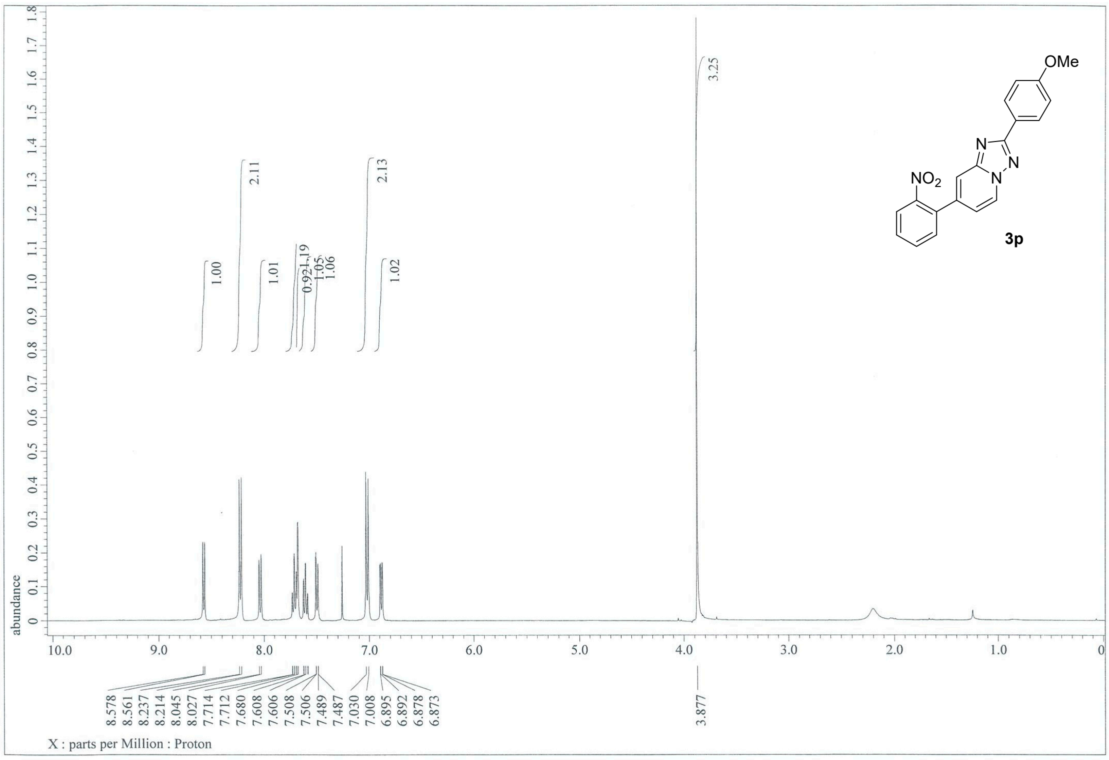

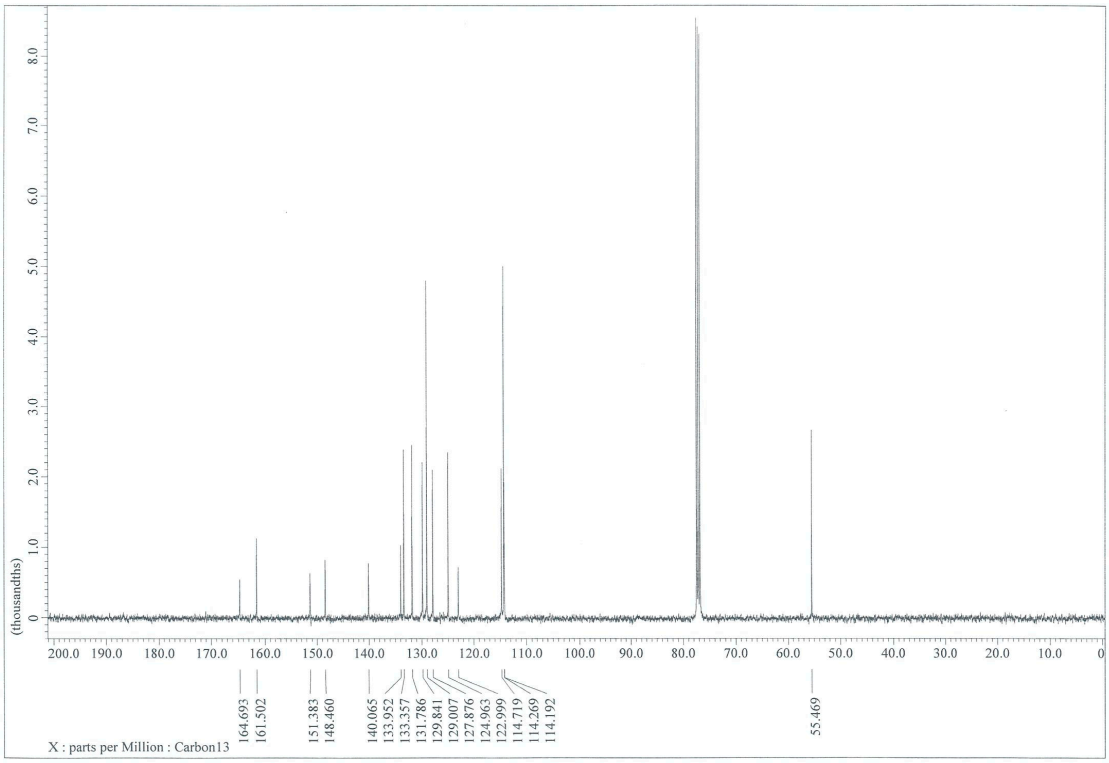

^{1}H (400 MHz, $CDCl_3$) and ^{13}C NMR (100 MHz, $CDCl_3$) of 7-(4-fluorophenyl)-2-(4-methoxyphenyl)-[1,2,4]triazolo[1,5-*a*]pyridine (3q)

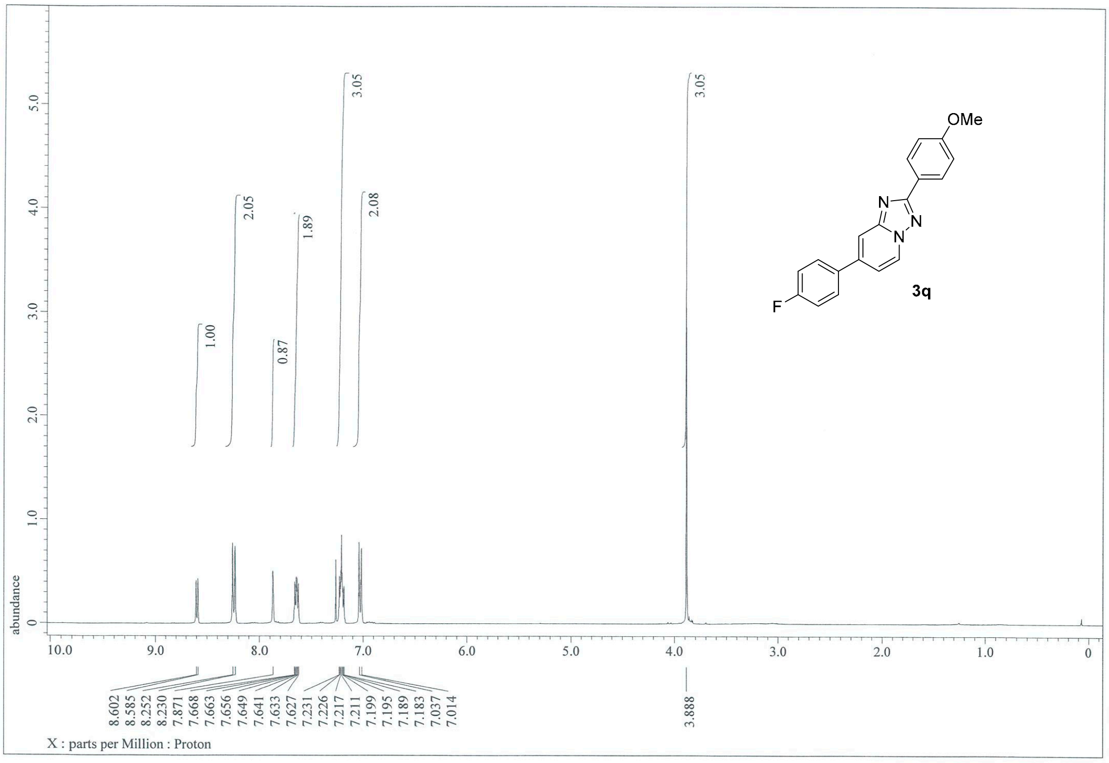

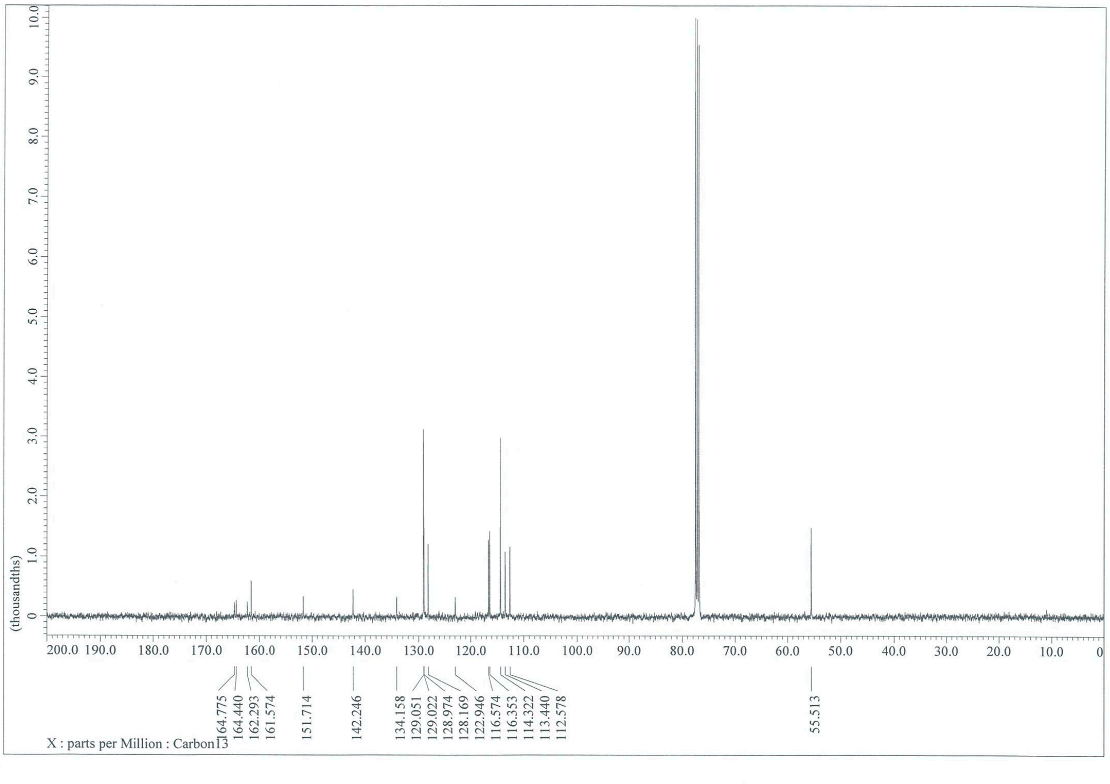

^{19}F NMR (376 MHz, $CDCl_3$) of 7-(4-fluorophenyl)-2-(4-methoxyphenyl)-[1,2,4]triazolo[1,5-*a*]pyridine(3q)

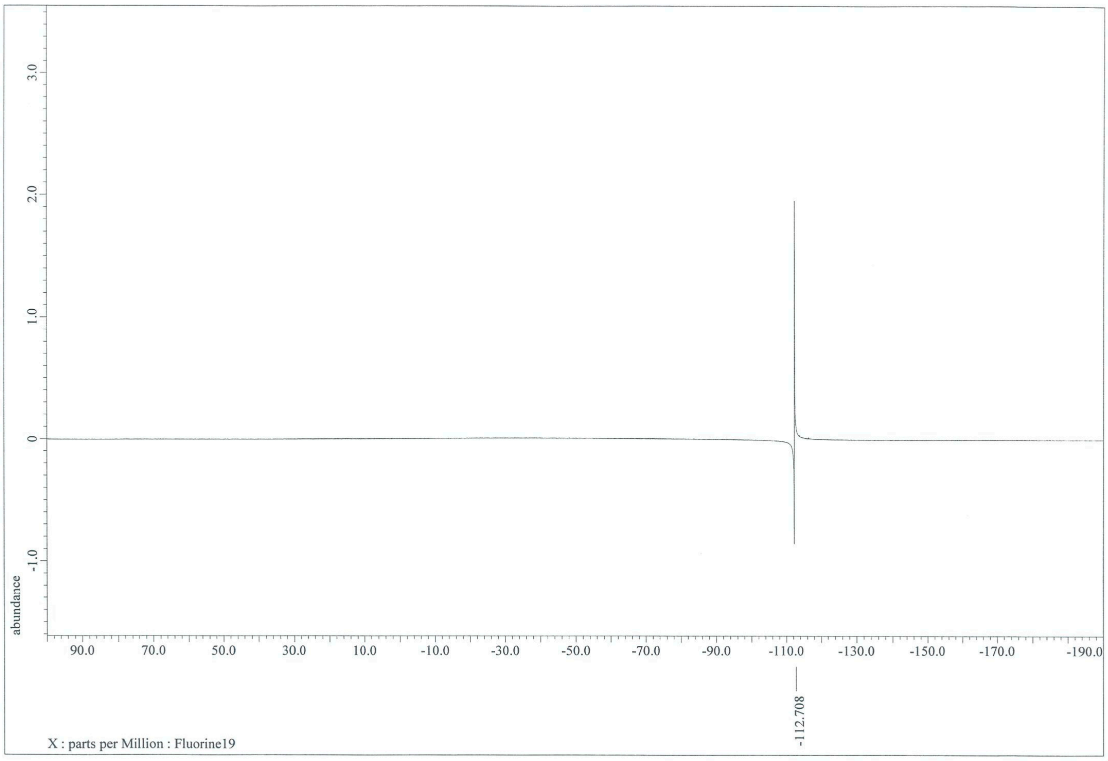

^{1}H (400 MHz, $CDCl_3$) and ^{13}C NMR (100 MHz, $CDCl_3$) of 7-(4-chlorophenyl)-2-(4-methoxyphenyl)-[1,2,4]triazolo[1,5-*a*]pyridine (3r)

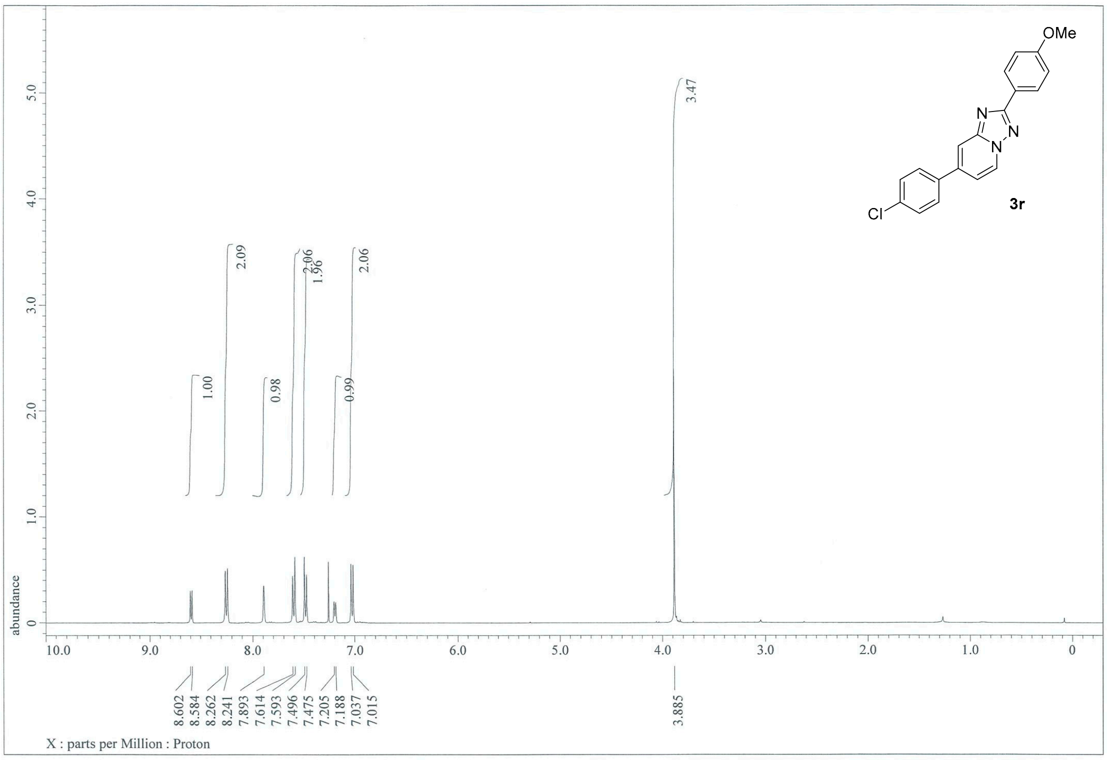

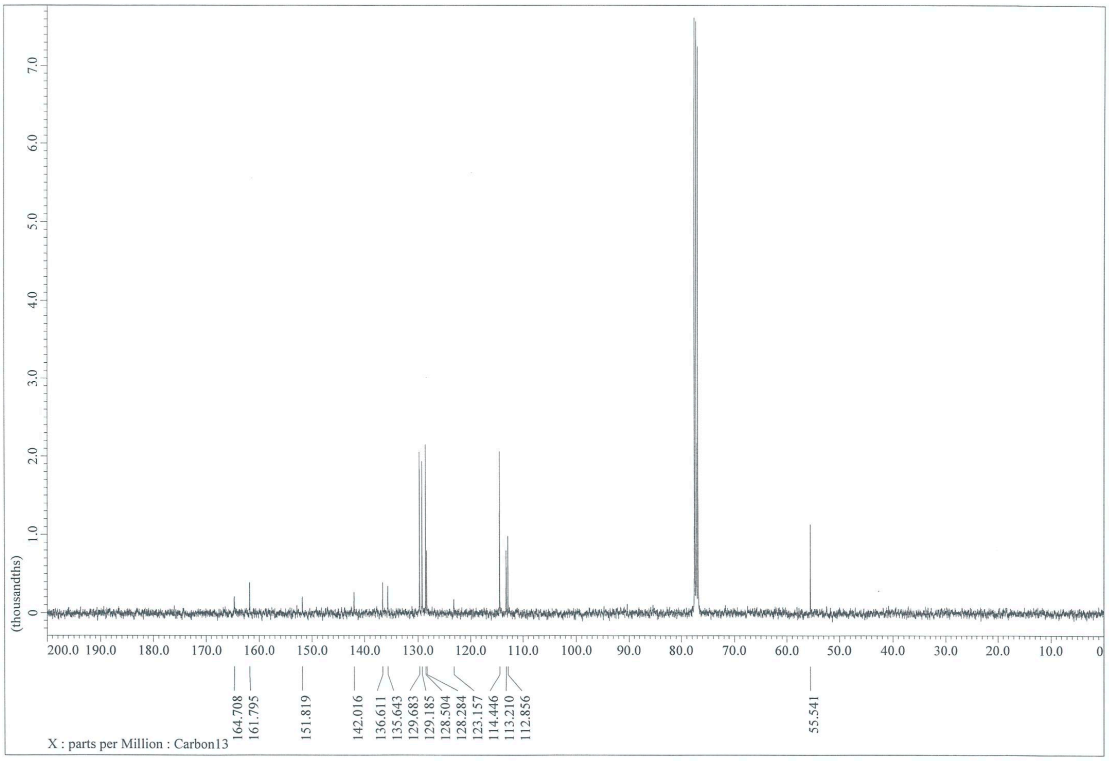

^1H (400 MHz, $CDCl_3$) and ^{13}C NMR (100 MHz, DMSO-d_6) of 7-(4-bromophenyl)-2-(4-methoxyphenyl)-[1,2,4]triazolo[1,5-*a*]pyridine (3s)

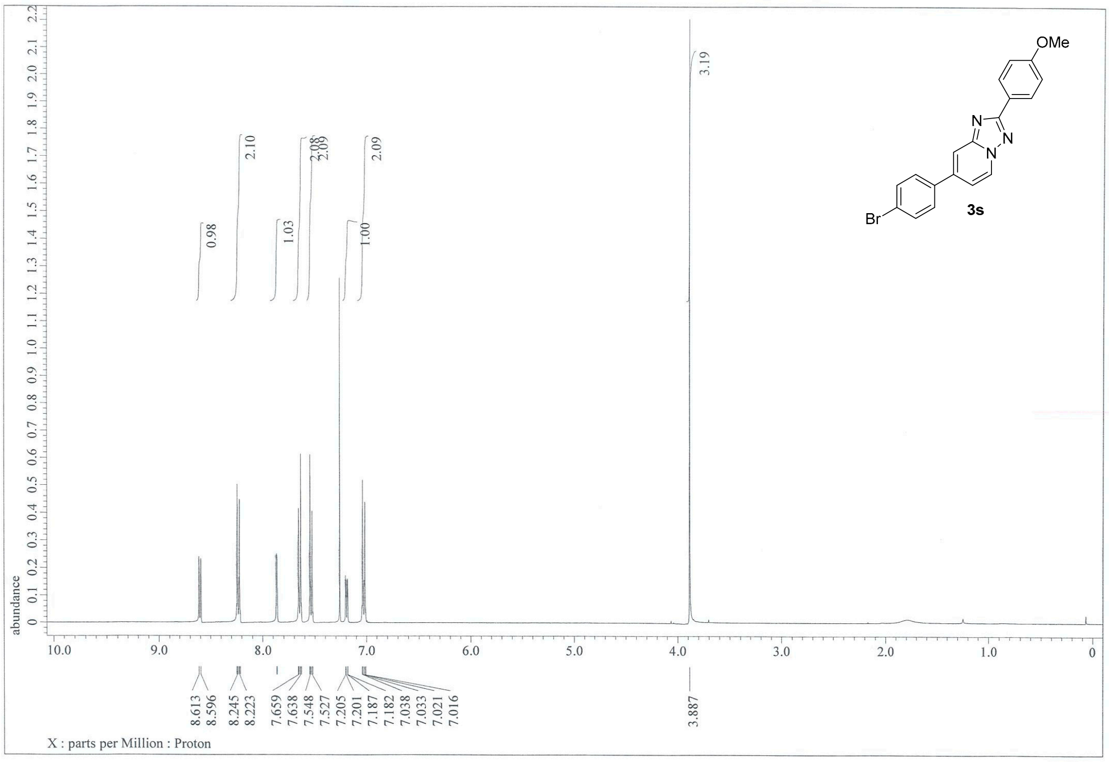

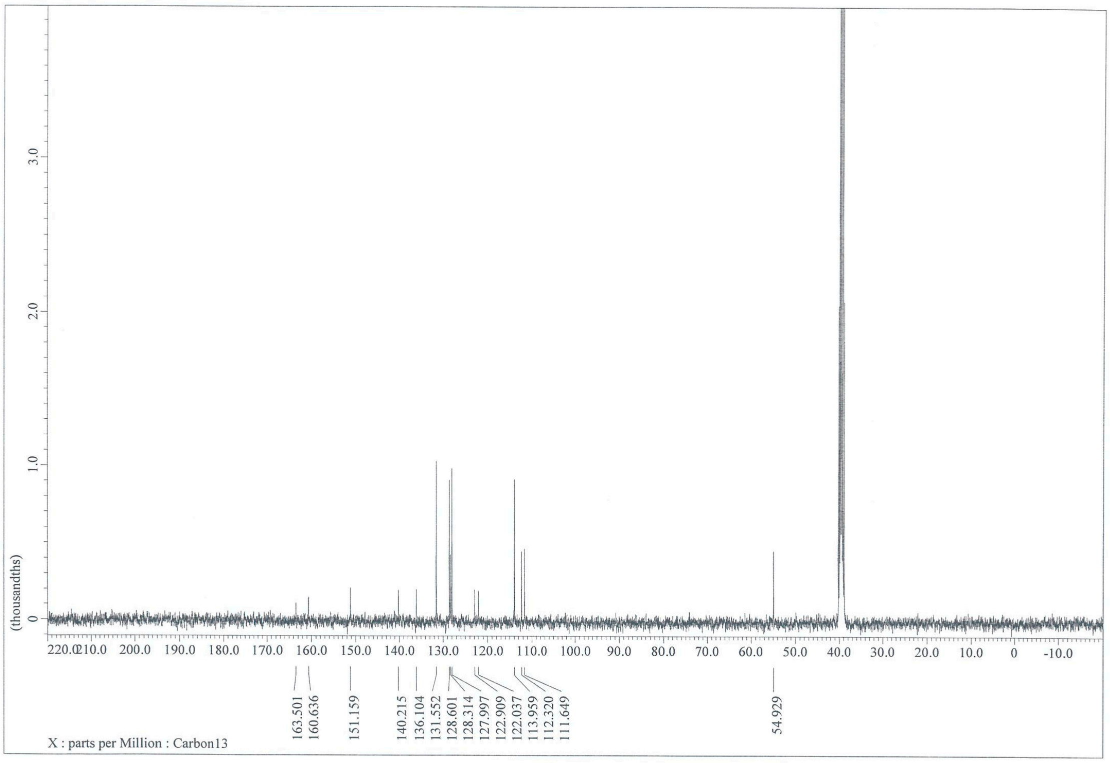

^{1}H (400 MHz, $CDCl_3$) and ^{13}C NMR (100 MHz, DMSO-d_6) of 7-(4-iodophenyl)-2-(4-methoxyphenyl)-[1,2,4]triazolo[1,5-*a*]pyridine (3t)

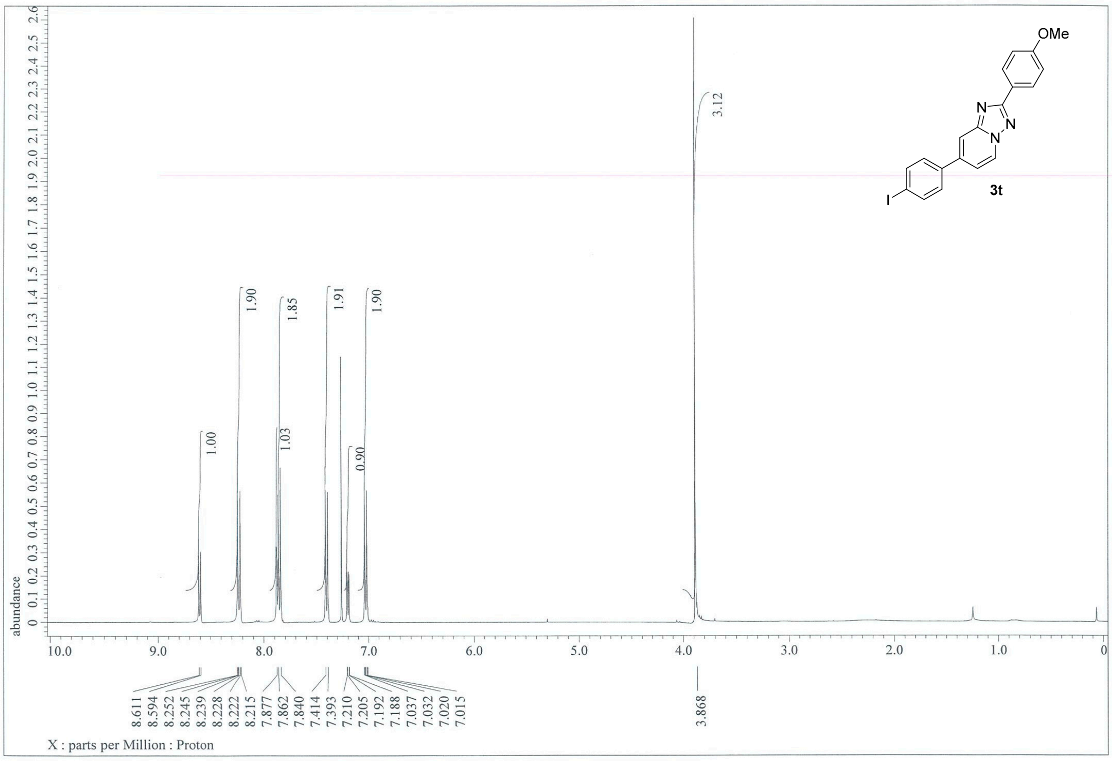

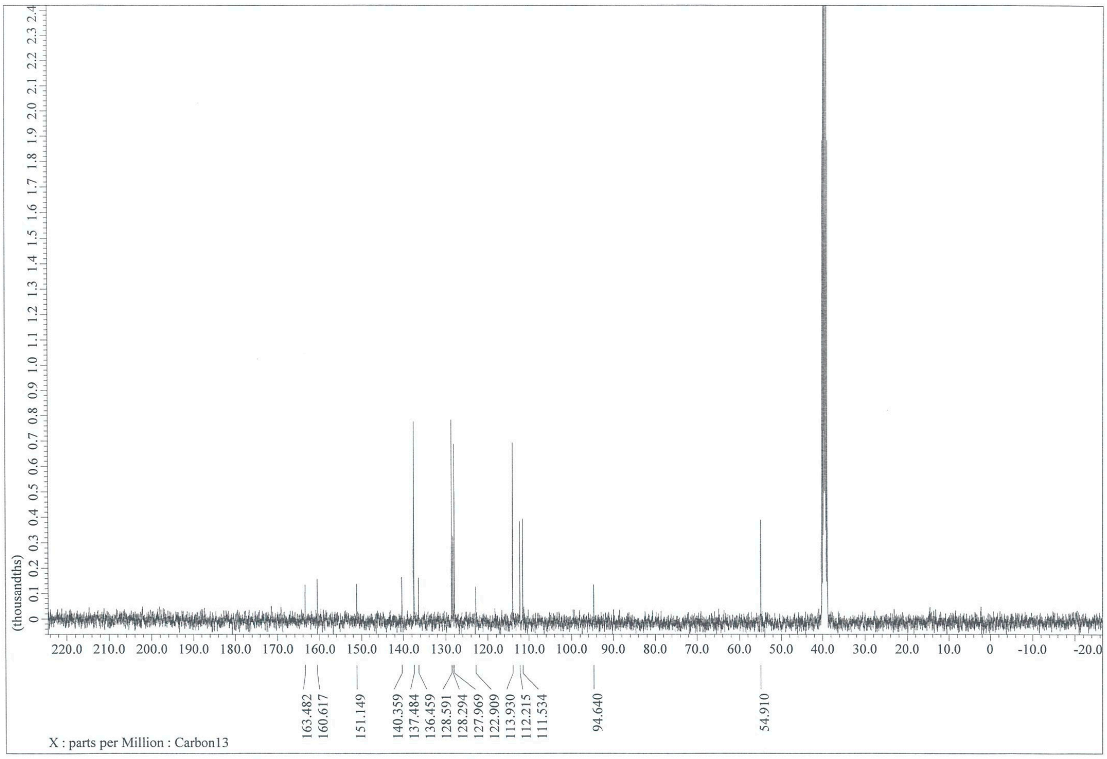

^{1}H (400 MHz, $CDCl_3$) and ^{13}C NMR (100 MHz, $CDCl_3$) of 7-(2,5-dichlorophenyl)-2-(4-methoxyphenyl)-[1,2,4]triazolo[1,5-*a*]pyridine (3u)

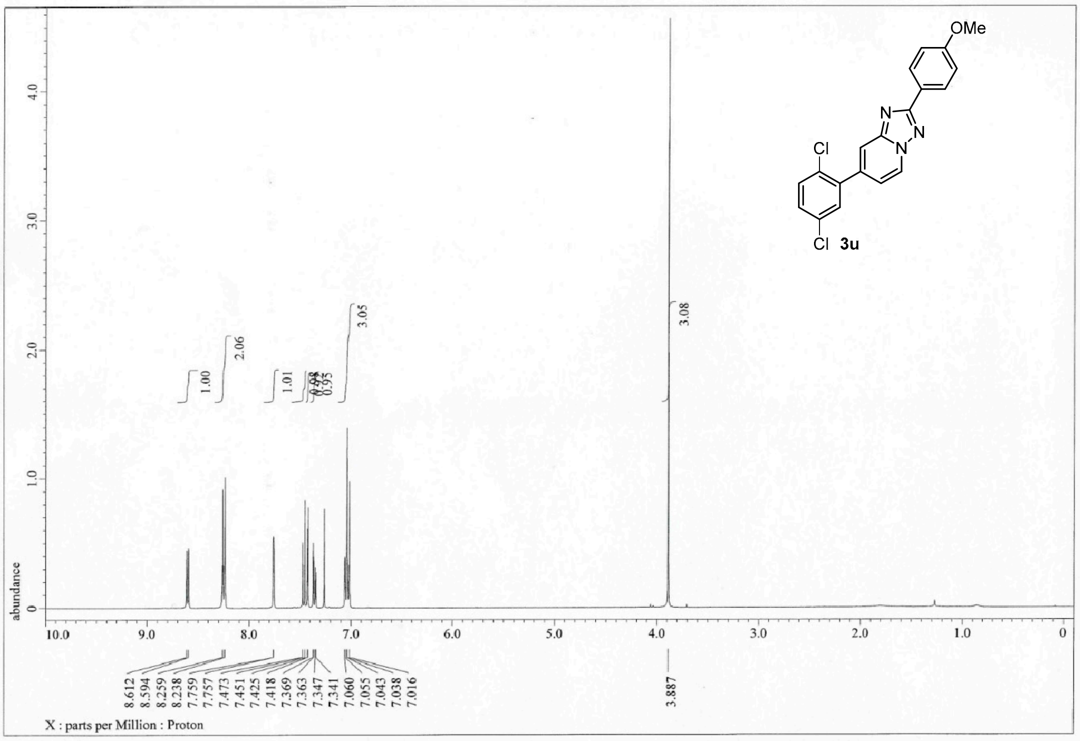

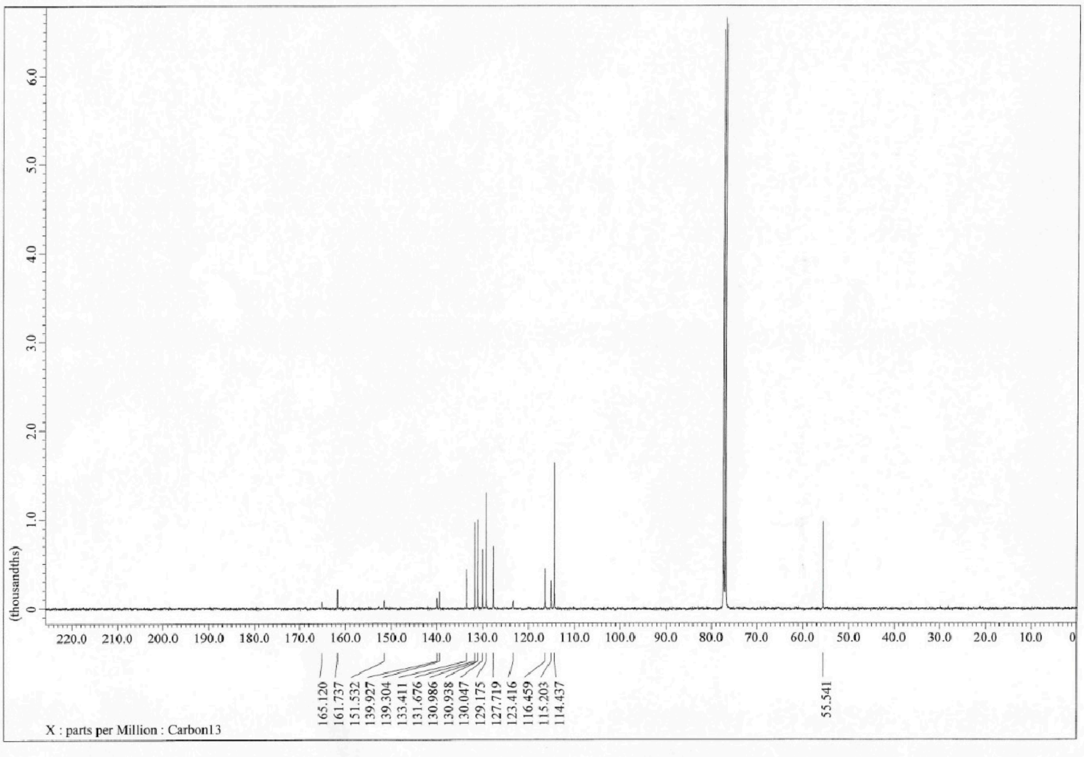

^{1}H (400 MHz, DMSO-*d$_6$*) $_3$) and ^{13}C NMR (100 MHz, DMSO-*d$_6$*) of 7-(4'-methoxy-[1,1'-biphenyl]-4-yl)-2-(4-methoxyphenyl)-[1,2,4]triazolo[1,5-*a*]pyridine (**5**), (both the NMR experiments were conducted at 413 K due to the of poor solubility of the **5**).

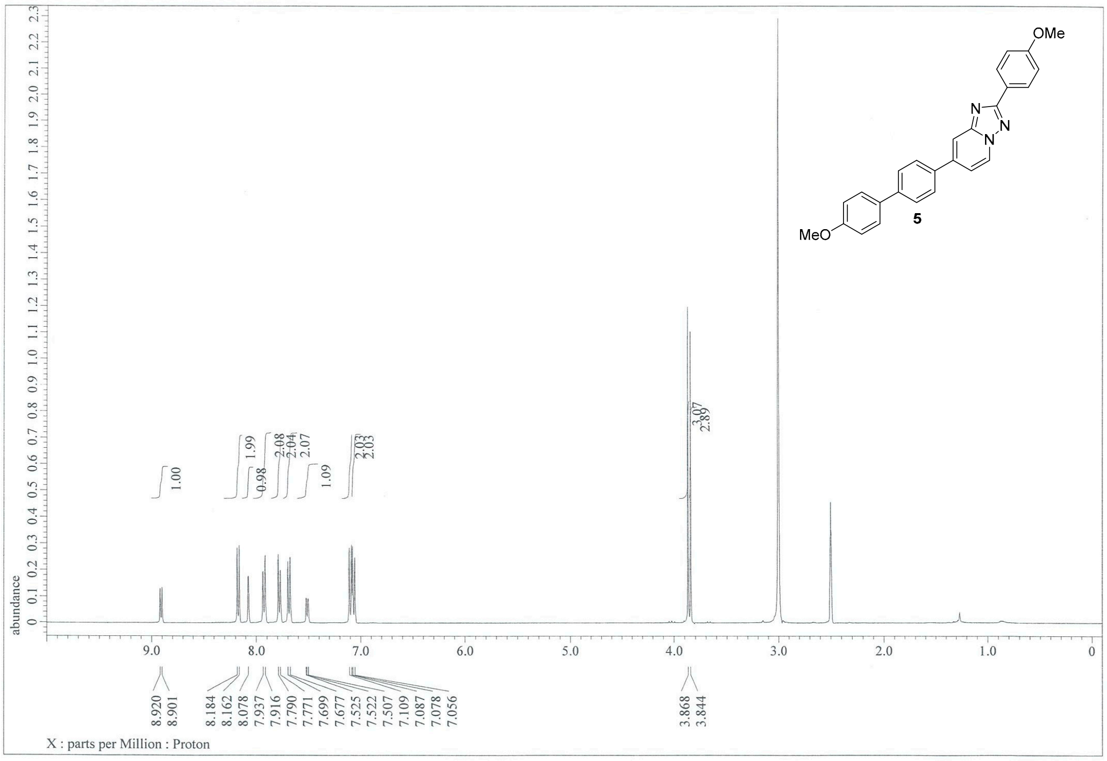

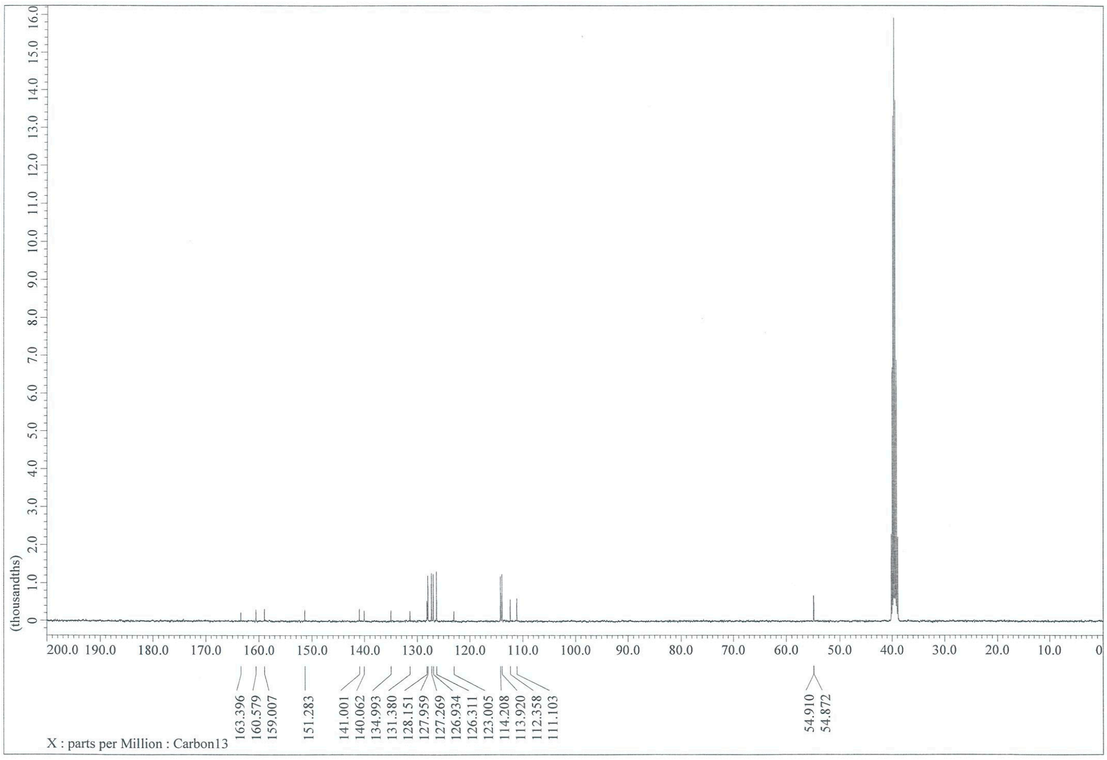

^{1}H (400 MHz, DMSO-*d$_6$*) 3) and ^{13}C NMR (100 MHz, DMSO-*d$_6$*) of 2-(4-methoxyphenyl)-7-(4-((4-methoxyphenyl)ethynyl)phenyl)-[1,2,4]triazolo[1,5-*a*]pyridine (**7**), (both the NMR experiments were conducted at 413 K due to the of poor solubility of the **7**).

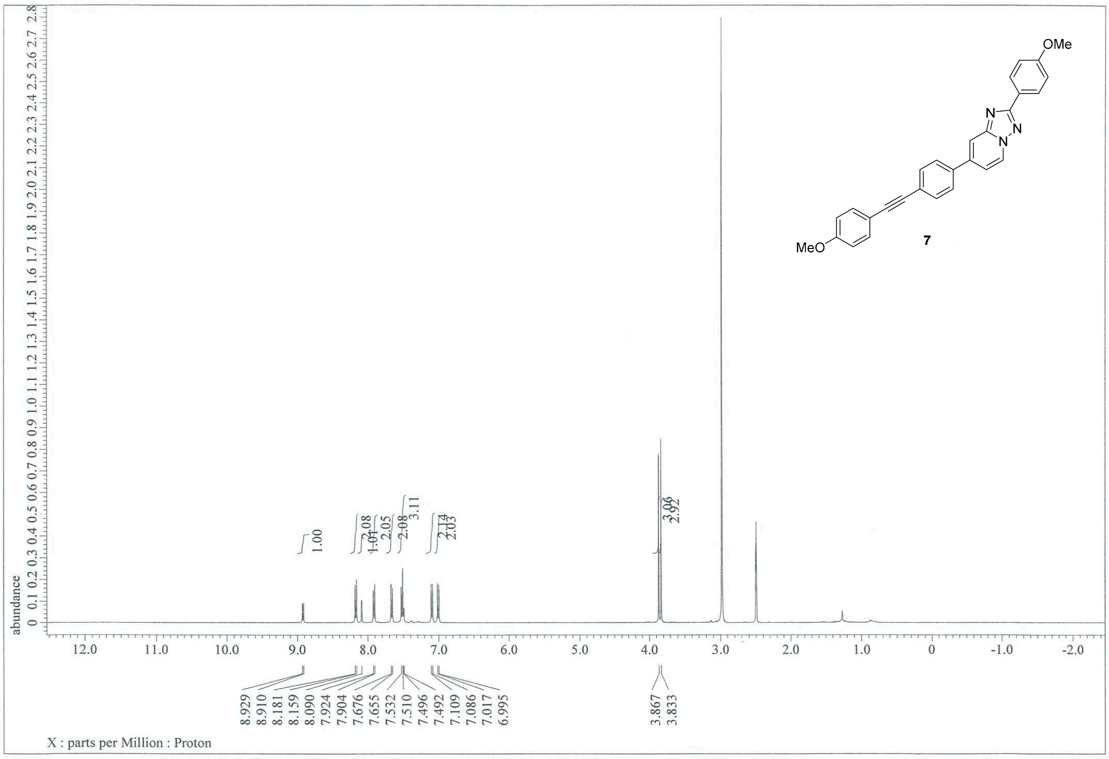

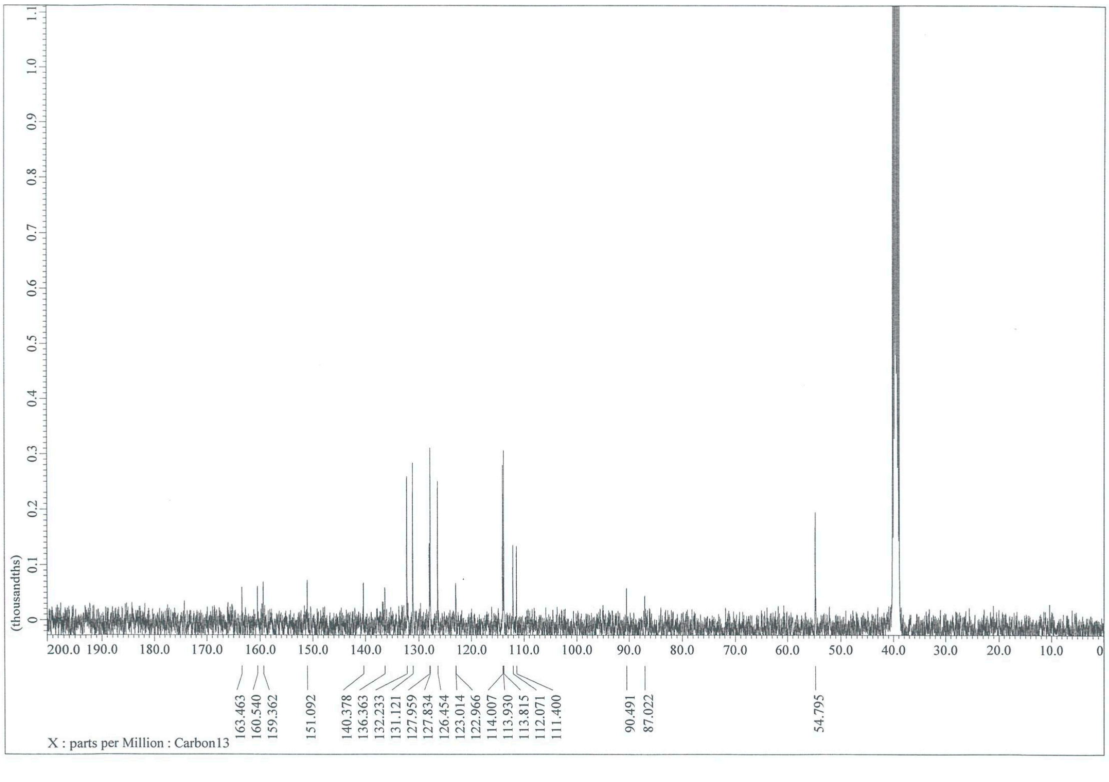

Supplement: Supplementary file 1 [file molecules-29-00894-s001.zip › molecules-2847251-supplementary.pdf]
